# Beyond single-use: a systematic review of environmental, economic, and clinical impacts of endoscopic surgical instrumentation

**DOI:** 10.1097/JS9.0000000000002141

**Published:** 2024-11-18

**Authors:** Myrthe M.M. Eussen, Martine Moossdorff, Lianne M. Wellens, Philip R. de Reuver, Tim Stobernack, Leon Bijlmakers, Merel L. Kimman, Nicole D. Bouvy

**Affiliations:** a Department of Surgery, Maastricht University Medical Center, Maastricht; b NUTRIM School of Nutrition and Translational Research in Metabolism, Maastricht University, Maastricht; c Department of General Practice, Amsterdam University Medical Center, Amsterdam; d Department of Surgery, Radboud University Medical Center, Nijmegen; e Department of Intensive Care Medicine, Radboud University Medical Center, Nijmegen; f IQ Health science department, Radboud University Medical Center, Nijmegen; g Department of Clinical Epidemiology and Medical Technology Assessment (KEMTA), Maastricht University Medical Centre, Maastricht, The Netherlands

**Keywords:** contamination, economic, endoscopic surgery, environmental, instruments

## Abstract

**Background:**

The evolution of endoscopic surgery has introduced a multitude of instruments, available in both disposable and reusable variants, influencing practices across various surgical specialties. Instrument selection is complex, considering individual preferences and institutional factors such as costs, instrument performance, and factors related to cleaning and sterilization. Notably, environmental sustainability has gained prominence due to the threat of climate change. This review assessed the existing literature to facilitate evidence-informed decision-making, encompassing clinical and economic efficacy, environmental friendliness, and other important criteria.

**Materials and Methods:**

Following PRISMA guidelines, searches were conducted in Pubmed, Embase, Web of Science, and The Cochrane Library for studies comparing the environmental impact, costs, instrument performance, and contamination risk of disposable versus reusable instruments or new versus reprocessed disposables in endoscopic surgery. Life-Cycle Assessments (LCAs) were included to quantify the climate impact. Exclusions included veterinary studies, general endoscopic procedures, and novel instruments.

**Conclusion:**

The search yielded 15 809 studies, 53 studies meeting the inclusion criteria: 38 compared disposable versus reusable instruments and 15 examined new versus reprocessed disposables. Reusables and/or reprocessed disposables showed favorable environmental and economic outcomes compared to new disposables. Instrument performance was comparable between the two groups. No studies were identified that investigated the clinical implications of contamination risk of disposables versus reusables. Six studies evaluating the contamination risk of reusables and reprocessed disposables showed residual pollution after cleaning and sterilization, although data on clinical outcome lacked.

**Interpretation:**

This review underscores the environmental benefits of reusables and favors both reusable and reprocessed disposables for their economic advantages. The lack of clear evidence favoring one type over the other in instrument performance necessitates further research. Addressing contamination risks requires additional studies on the clinical impact of residual substances. Future research should report outcomes on environmental sustainability, costs, instrument performance, and contamination risk.

## Introduction

Highlights

- Environmental sustainability should be central in instrument selection.
- Fifty-three studies comparing disposable vs. (new and reprocessed) reusable instruments.
- Findings reveal a significant environmental advantage for reusable instruments.
- Economic analysis favors reusable and reprocessed disposables.
- Reusables and reprocessed disposables maintain performance and safety standards.

Endoscopic surgery has experienced a transformative revolution, characterized by the development of innovative instruments like staplers and electrocautery tools. These advancements have led to significant patient benefits, including faster recovery, reduced postoperative pain, and improved cosmetic outcomes, making endoscopic surgery has become a cornerstone across various medical specialties^1^.

Instruments for endoscopic surgery are available in both disposable and reusable versions. Disposables gained prominence due to their ability to prevent cross-infection^1^. Reusables require cleaning and sterilization, introducing a risk of contamination^1–6^. Surgical site infections (SSIs) affect 1–16% of cases, leading to complications like reoperations and longer hospital stays^7,8^. While patient safety is crucial, factors like cost, availability, surgeon preference, and sustainability also influence this choice^9^.

The need for sustainability in healthcare arises from understanding its societal and public health consequences^10,11^. Research has suggested that per capita greenhouse gas (GHG) emissions are higher in the global North, placing a disproportionate burden of the consequences on the global South^10,11^. This underscores the need for clinicians, especially in resource-intensive producing settings, to anticipate adverse health conditions caused by global warming and climate change. The Lancet and the WHO have identified climate change as the paramount threat confronting humanity and public health^10,11^.

The healthcare sector’s climate footprint accounts for 4·4% of global net emissions, with developed countries exhibiting emissions ranging from 6 to 8%^12^. Surgical practices generate 20–33% of these emissions^13–15^. Life-cycle assessments (LCAs), which measure the environmental impact of a product, show that surgical disposables contribute substantially to emissions. Comes *et al*. (2024) found that surgical disposables contribute to 40% of the total CO_2_ equivalent (CO_2_eq) for a laparoscopic cholecystectomy, while switching to reusables can reduce emissions by 40–66%, offering a more sustainable option for surgery^15–17^.

In response to the climate change, organizations have formulated strategies, such as the United Nations’ 2030 Agenda for Sustainable Development Goals, the Paris Agreement and The European Green Deal^18,19^. Many countries have adopted Green Deal programs to integrate sustainable practices within healthcare, reflecting a commitment to align healthcare with climate goals.

Considering these developments to combat climate change, healthcare must shift towards environmental sustainability. With disposable instruments contributing to emissions, reducing their use becomes a pivotal strategy for waste reduction. However, the choice for disposables, often driven by several concerns, reflects a hesitancy that may come at the expense of our planet. A comprehensive evaluation encompassing the environmental impact, costs, instrument performance, and contamination risk is essential for informed decision-making.

The aim of this review is to provide an overview of the existing literature on these decision-making aspects. By weighing advantages and disadvantages of disposable versus reusable and new versus reprocessed endoscopic instruments, this review will facilitate decision-making that prioritizes both clinical efficacy, economic considerations, infection control, and environmental responsibility.

## Methods

### Study protocol and registration

This systematic review adhered to the 2020 preferred reporting items for systematic reviews and meta-analyses (PRISMA) (Supplemental Digital Content 1, http://links.lww.com/JS9/D560) and assessing the methodological quality of systematic reviews (AMSTAR) (Supplemental Digital Content 2, http://links.lww.com/JS9/D561) guidelines^20,21^. The study protocol was registered at PROSPERO.

### Search strategy

The search was conducted in September 2023 and aimed to identify relevant studies published across Pubmed, Embase, Web of Science, and The Cochrane Library. The search strategy comprised four key components: 1) endoscopic surgical procedures, 2) reusable instruments, 3) disposable instruments, and 4) four outcome measures (environmental sustainability), costs, instrument performance, and contamination risk. Databases were searched using MeSH, Emtree, and free terms combined through AND-OR combinations. No time restriction was applied. The complete search strategy is available in Appendix A (Supplemental Digital Content 3, http://links.lww.com/JS9/D562). A manual search of systematic reviews’ reference lists was performed to identify additional literature.

### Selection process

Eligibility criteria included studies presenting original data that compared disposable and reusable or new and reprocessed disposable instruments in human endoscopic surgery. Animal studies for veterinary purpose, nonendoscopic surgery, general endoscopic procedures, and studies on novel instruments not in standard practice were excluded as this review focuses on current clinical practices and offers practical guidance for sustainable change. Secondary sources such as technical descriptions, letters to the editor, conference proceedings, and commentaries were not considered. Studies published in languages other than English, Dutch, French, or German were excluded.

After removing duplicates, potentially eligible studies underwent independent screening by two reviewers (M.E. and M.M.) based on title and abstract, with discrepancies resolved through consensus meetings. Studies meeting eligibility criteria after full-text screening were included, and the EndNote Reference Management Tool facilitated reference management.

### Data extraction

Two reviewers (M.E. and M.M.) independently extracted information from each article, including author, year of publication, country, study period, type and quantity of investigated instruments, number of instrument reuses, surgical procedure, primary aim, and study findings. Separate tables were created for studies comparing disposable and reusable items and those comparing new disposables with reprocessed ones. Data specific to the type of outcome, such as CO_2_ equivalent or costs, was extracted (see Table B1-4 in Appendix B, Supplemental Digital Content 4, http://links.lww.com/JS9/D563). To account for variations in study years and different currencies, the impact on costs was presented as relative cost reductions, extracted from studies or manually calculated by the two reviewers (M.E. and M.M.).

### Risk of bias assessment

Two reviewers (M.E. and M.M.) independently assessed the risk of bias of the included studies, using diverse metrics. Life-Cycle Assessments (LCAs), according to ISO 14040, 14044-guidelines, or the Greenhouse Gas protocol, were appraised using modified criteria adapted from Drew *et al*., drawing on Weidema’s Guidelines for Critical Review of LCAs^22,23^. Nonrandomized studies underwent assessment using the Cochrane ROBINS-I tool, for randomized studies the Risk of Bias 2 (RoB 2) tool was used, and for animal studies the SYRCLE risk of bias tool^24–26^. An arbitrator (L.W.) resolved discrepancies between the two reviewers (M.E. and M.M.) during the study selection and assessment process.

### Analysis

Data from the included studies were synthesized in tables, with findings explicated through a narrative approach. Due to significant heterogeneity among the included studies, conducting a meta-analysis was deemed unfeasible.

## Results

### Study selection

**Figure 1:**
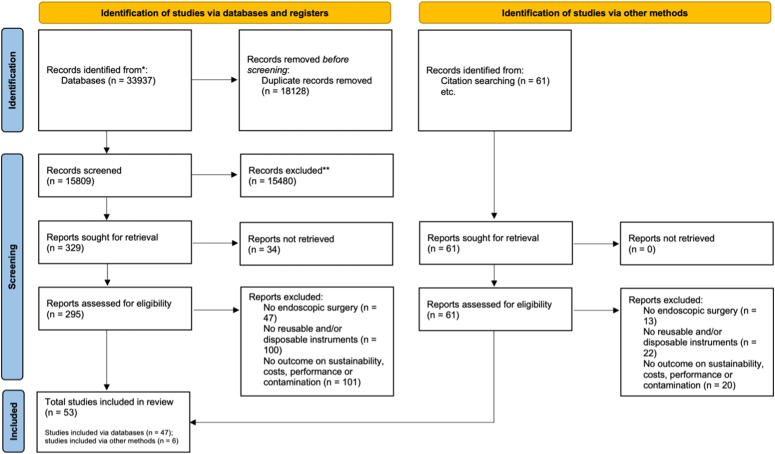
The PRISMA flowchart outlining the strategy for study selection.

The search yielded 15 809 studies after removing duplicates. Initial screening of titles and abstracts identified 329 potential studies. Full-text assessment was not possible for 34 studies, mainly due to language restrictions, leaving 295 studies eligible for further consideration. Reference checking added 61 studies, resulting in a total of six additional included studies. Ultimately, 53 studies were incorporated into this review (see Fig. 1).

### Study characteristics

Among the 53 studies, 38 studies investigated disposable versus reusable instruments in laparoscopic surgery (see Table 1A), and the remaining 15 studies explored new versus reprocessed disposables in general laparoscopic surgery (see Table 1B). Due to the diversity in study designs, a heterogeneity was observed among the included studies (see Table 1A).

**Table 1A:** Characteristics of included studies comparing disposables with reusables (*n*=38).

Author	Year	Country	Inclusion period	Study type	Type of instrument (No. per procedure)	Aim of the study
Bariatric surgery (*n*=2)						
Meissner *et al*.^1^	2023	Germany	2022	PS	D: Echelon Flex stapler with staple line reinforcement (1)	Compare the environmental outcomes of disposable and reusable staplers for SG and RYGB
					R: Signia stapler with reinforced reloads (1)	
Yung *et al*.^2^	2010	USA	NA	PS	D: Olympus Sonosurg scissors	Compare economic outcomes and performance of disposable and reusable ultrasonic shears for LBS^a^
					R: Ethicon Harmonic Scalpel	
General surgery (*n*=26)						
Adler *et al*.^3^	2005	Germany	NA	PS	D: trocars (4), scissors (2) and Veress cannula (1)	Compare economic outcome and environmental impact of disposable and reusable instruments for LC
					R: all other instruments for LC	
Alfa *et al*.^4^	2003	Canada	NA	IVS	Ported devices: peritoneal scissors (2), forceps (Alligator, Dolphin dissection, Debakey) (4)	Evaluate decontamination (in terms of cleaning efficacy) of ported and nonported instruments for GLP
					Nonported devices: scissors (1), forceps (Dolphin, DeBakey) (2)	
Apelgren *et al*.^5^	1993	Canada	NA	PS	D: Veress needle (1), 5 mm trocar (2), 10 mm trocar (2), reducers, clip appliers (2) with package of clips (2)	Compare economic outcome of disposable and reusable instruments for LC
					R: Veress needle (1), 5 mm trocar (2), 10 mm trocar (2), reducers, clip appliers (2) with package of clips (2)	
Baxter *et al*.^6^	2006	UK	NA	PS	Three different trays for laparoscopic surgery	Evaluate decontamination (in terms of proteinaceous contamination after cleaning and sterilization) of reusable instruments for GLP
Boberg *et al*.^7^	2022	Sweden	NA	LCA and LCC	D: 5–12 mm trocar (2) and 5 mm trocar (2)	Compare economic outcome and environmental impacts of disposable and reusable trocars for LC
					R: 12 mm trocar (1), 5 mm trocar without stopcock (1), 5 mm trocar with stopcock (1)	
Demoulin *et al*.^8^	1996	Belgium	1995	PS	D: Veress needle (1), 5 mm trocars (2), 10 mm trocars (2), reducer (1), scissors (1), dissector (1), 5 mm grasper (1), 10 mm grasper, clip-applier with clips (1), suction (1), L-hook electrode (1)	Compare economic outcome of disposable and reusable instruments for LC
					R: Veress needle (1), 5 mm trocars (2), 10 mm trocars (2), reducer (1), scissors (1), dissector (1), 5 mm grasper (1), 10 mm grasper, clip-applier with clips (1), suction (1), L-hook electrode (1)	
DesCôteaux *et al*.^9^	1998	Canada	1995–1996	PS	D: limited-reusable scissors (NA)	Compare economic outcomes and performance of limited-reusable and reusable instruments for GLP
					R: scissors (NA)	
					D: limited-reusable hook cautery (NA)	
					R: hook cautery (NA)	
Eddie *et al*.^10^	1996	Australia	1991–1993	RS	D: trocars (3-5), scissors (NA), clip applicators (NA) and graspers (NA)	Compare economic outcome of disposable and reusable instruments for different laparoscopic procedures^b^
					R: trocars (3-5), scissors (NA), clip applicators (NA) and graspers (NA)	
Fengler *et al*.^11^	1998	Germany	1990–1996	PS	D: scissors (1), forceps (3), trocars (4), Veress needle	Compare economic outcome of disposable and reusable instruments for GLP
					R: scissors (1), forceps (3), trocars (4), Veress needle	
Fengler *et al*.^12^	2000	Germany	NA	PS	Three different trays for laparoscopic surgery including straight scissors (NA), straight traumatic and atraumatic grasping forceps (NA), monopolar hooks (NA) and grasping forceps (NA)	Evaluate decontamination (in terms of proteinaceous contamination after cleaning and sterilization) of reusable instruments for GLP
Harper *et al*.^13^	2009	USA	NA	AS	D: Direct Drive (Applied medical)	Compare the durability and ergonomics of laparoscopic scissors for GLP
					D: Endopath (Ethicon Endosurgery)	
					R: Auto Suture Endo Shear (U.S. Surgical)	
Ibbotson *et al*.^14^	2013	Germany	NA	LCA and LCC	D: plastic scissors (2)	Compare economic outcome and environmental impacts of disposable and reusable scissors
					D: stainless steel scissors (2)	
					R: stainless steel scissors (2)	
Kelty *et al*.^15^	2000	UK	NA	IVS^c^	D: 10 mm trocars (NA)	Compare the ergonomics of disposable and reusable trocars for GLP
					R: 10 mm trocars (NA)	
Klar *et al*.^16^	2011	Germany	NA	AS	D: LigaSure (Covidien-Valleylab) (NA)	Compare performance of disposable and reusable vessel-sealing device for GLP
					R: MarSeal (KLS Martin) (NA)	
MacFadyen *et al*.^15^	1994	USA	1993	PS	D: surgical instruments (NA)	Compare economic outcome of disposable and reusable surgical instruments for LC
					R: surgical instruments (NA)	
Mahmoud *et al*.^17^	2019	Egypt	2016–2017	PS	D: harmonic scalpel (Harmonic, Ethicon Endo-surgery Inc.) (1)	Compare economic outcomes and performance of disposable and reusable energy devices for LC
					R: monopolar electrocautery (hook, grasper, scissors) and clips (NA)	
Manatakis *et al*.^18^	2014	Greece	2012–2013	RS	D: curved monopolar dissector (1), atraumatic grasp monopolar forceps with ratchet (2), Metzenbaum monopolar scissors (1), suction (1), Veress needle (1), Hasson cannula (1), 5 mm trocars (2), 12 mm trocars (2)	Compare economic outcome of disposable and reusable surgical instruments for GLP
					R: curved monopolar dissector (1), atraumatic grasp monopolar forceps with ratchet (2), Metzenbaum monopolar scissors (1), suction (1), Veress needle (1), Hasson cannula (1), 5 mm trocars (2), 12 mm trocars (2)	
Montero *et al*.^19^	2010	USA	2008	IVS^d^	D: L-hook (Covidien, Conmed and Megadyne) (NA)	Compare performance (in terms of insulation failure) in disposable and reusable instruments for GLP
					R: laparoscopic instruments (NA)	
					R: instrument set (NA)	
Murdoch *et al*.^20^	2006	UK	NA	PS	retractors (NA), scissors (NA), forceps (NA), gags (NA), birkett (NA), dissectors (NA)	Evaluate decontamination (in terms of proteinaceous contamination after cleaning and sterilization) of instruments for GLP
Nezhat *et al*.^21^	1991	USA	1990	PS	D: shielded trocars (Ethicon)	Compare performance of disposable and reusable trocars for GLP
					R: conventional trocar	
Owusu *et al*.^22^	2022	Ghana	NA	PS	dissecting forceps (NA), kocher forceps (NA), rampley sponge holding forceps (NA), curved mosquito artery forceps (NA), metzenbaum scissors (NA), mayo scissors (NA), Langenberg retractor (NA), deaver retractor (NA)	Evaluate decontamination (in terms of bacterial contaminants) of instruments for GLP
Raina *et al*.^23^	2018	India	2015–2016	PS	Instruments for laparoscopy	Evaluate decontamination (in terms of postoperative port-site infections) of instruments for GLP^e^
Richter *et al*.^24^	2006	Germany	NA	AS	D: LigaSure (NA)	Compare performance (in terms of vessel sealing devices) in disposable and reusable instruments for GLP
					R: BiClamp (NA)	
Rizan *et al*.^25^	2022	UK	NA	LCA & LCC	D: 5 mm trocar (2), 11 mm trocar (2), laparoscopic scissor (1), clip applier with cartridge (1)	Compare economic outcomes and environmental impact of disposable and reusable instruments for LC
					R: 5 mm reusable trocar and cannula with disposable valve (2), 10 mm reusable trocar and cannula with disposable valve (2), reusable handle without ratchet with disposable Metzenbaum scissors (1), reusable multi-fire clip applier 10 mm with disposable cartridge (1)	
Rizan *et al*.^26^	2023	UK	2019–2020	LCA	D: 5 mm trocar (NA), 12 mm trocar (NA), laparoscopic scissors (NA), clip applier (NA)	Evaluate environmental impact of disposable and reusable products used within five common procedures (s.a. LC)
					R: Hopkins laparoscope (NA), Kelly crocodile grasping forceps (NA), Langenbeck retractor small (NA), Raptor toothed grasping forceps (NA), Rampley sponge holder forceps (NA)	
Slater *et al*.^27^	2009	UK	2001–2007	PS	D: 5 mm trocars (NA), 12 mm trocars (NA) with sleeves, clip applicator with hemo-o-lok clips (NA), Veress needle (NA)	Compare economic outcome of disposable and reusable instruments for LC
					R: 5 mm trocars (2), 10 mm trocars (2) with sleeves (4), clip applicator with hemo-o-lok clips (NA), Veress needle (NA)	
Gynecological surgery (*n*=7)						
Hasanov *et al*.^28^	2017	Germany	2014–2016	RCT	D: 5 mm Ligasure	Compare economic outcomes and performance of disposable and reusable vessel-sealing devices for LH (only benign conditions)
					R: 5 mm Marseal IQ	
Holloran-Schwartz *et al*.^29^	2016	USA	2013	RCT	D: Ligasure 5 mm Blunt Tip (1)	Compare economic outcomes and performance of disposable and reusable energy devices for LH
					R: Rotating bipolar forceps with 5 mm monopolar scissors (1)	
Martínez-Zamora *et al*.^30^	2009	Spain	2007–2008	RCT	D: GyneCare Morcellex	Compare economic outcomes and performance of disposable and reusable electric morcellators for LH and LM
					R: VarioCarve	
Ransom *et al*.^31^	1996	UK	1988–1994	RS	D: 10 mm infraumbilical cannula	Compare economic outcomes and performance of disposable and reusable cannulas for GL
					R: 10 mm infraumbilical cannula	
Rothmund *et al*.^32^	2013	Germany	NA	RCT	D: EnSeal (Ethicon Endo-Surgery GmbH)	Compare performance of disposable and reusable sealing devices for LASH (only benign conditions)
					R: Bipolar RoBi clamp (Karl Storz)	
Schaer *et al*.^33^	1995	Switzerland	1993–1994	PS	D: optics (NA), 5 mm trocar (NA), 10 mm trocar (NA), 12 mm trocar (NA), Veress needle (NA), converter (NA), graspers (NA), hook scissors (NA), microscissors (NA), suction (NA)	Compare economic outcome of disposable and reusable instruments for GL
					R: optics (1), Veress needle (1), 5 mm trocar (1), 10 mm trocar (1), probe (1)	
Thiel *et al*.^34^	2018	USA	2015–2016	LCA	D: laparoscopic instruments	Evaluate environmental impact of disposable and reusable instruments for LH
					R: laparoscopic instruments	
Pediatric surgery (*n*=2)						
Ebeid *et al*.^35^	2016	Egypt	2013–2015	PS	D: 5 mm trocars (2), 10 mm trocar (1)	Compare economic outcome of disposable and reusable trocars for PLA
					R: 5 mm trocars (2), 10 mm trocar (1)	
Graham *et al*.^36^	2019	USA	2011–2017	RS	D: 35 mm endoscopic stapler (1)	Compare economic and clinical outcomes, and environmental impacts of staples and clips for SIPESA
					R: nonabsorbable polymer Hem-o-lok clips (2) with reusable applier (1)	
Thoracic surgery (*n*=1)						
Weksler *et al*.^37^	2009	Brazil	2003–2007	RS	D: ultrasonic scalpel (Ethicon Endo-Surgery) (1)	Compare performance of disposable and reusable cautery devices for ETS
					R: 5 mm monopolar hook cautery (Edlo) (1)	

^a^ Laparoscopic gastric banding, biliopancreatic diversion with duodenal switch, gastric bypass, sleeve gastrectomy, revision.

^b^ Hernia repair, diagnostic laparoscopy, appendectomy, cholecystectomy, fundoplication, colorectal and duodenal ulcer.

^c^ Abdominal wall reconstruction by two plywood sheets with sheepskin in-between.

^d^ Insulation failure testing by using Mircomed PD-8K porosity detector.

^e^ Laparoscopic cholecystectomy (89.29%), laparoscopic appendectomy (6.25%), laparoscopic ovarian cystectomy (4·46%).

AS, animal study; D, disposable; ETS, endoscopic thoracic sympathectomy; GL, gynecologic laparoscopy; GLP, general laparoscopic practice; IVS, in-vitro study; LASH, laparoscopic supracervical hysterectomy; LBS, laparoscopic bariatric surgery; LC, laparoscopic cholecystectomy; LCA, life-cycle assessment; LCC, life-cycle costing; LH, laparoscopic hysterectomy; LM, laparoscopic myomectomy; NA, not available; PLA, pediatric laparoscopic appendectomy; PS, prospective study; R, reusable; RCT, randomized controlled trial; RS, retrospective study; RYGB, Roux-en-Y Gastric Bypass; SG, sleeve gastrectomy; SIPESA, Single-Incision Pediatric Endosurgery Appendectomy.

**Table 1B:** Characteristics of included studies comparing new with reprocessed disposables (*n*=15).

Author	Year	Country	Inclusion period	Study type	Type of instrument	Aim of the study
General surgery (*n*=15)						
Brady *et al*.^38^	2017	USA	2012–2015	PS	ND: LigaSure	Compare safety and economic outcomes of new disposable and reprocessed disposable bipolar energy devices for LCO
					RD: LigaSure	
Carungi *et al*.^39^	NA	USA	NA	IVS	ND: Harmonic scalpels	Compare safety and performance of new disposable and reprocessed disposable Harmonic scalpels for GLP
					RD: Harmonic scalpels	
Chan *et al*.^40^	2000	China	NA	IVS	RD: 10/11 mm trocars	Evaluate decontamination of reprocessed disposable trocars for GLP
Chivukula *et al*.^41^	2020	USA	NA	PS	ND: LigaSure	Compare decontamination of new disposable and reprocessed disposable bipolar energy devices for GLP
					RD: LigaSure	
Colak *et al*.^42^	2004	Turkey	2002	PS	ND: trocars, dissectors, curved scissors, jaws, graspers, hooks, clips	Compare safety and economic outcomes of new disposable and reprocessed disposable instruments for LC
					RD: trocars, dissectors, curved scissors, jaws, graspers, hooks, clips	
De Lion Botero Couto Lopes *et al*.^43^	2011	Brazil	NA	IVS	R: 5 mm grasper with ratchet handle, 5 mm curved scissors with monopolar cautery), 5 mm curved dissector with monopolar cautery, electrosurgery probe, Veress needle	Compare decontamination of reusable and reprocessed disposable instruments for GLP
					RD: 5 mm grasper with ratchet handle, 5 mm curved scissors with monopolar cautery), 5 mm curved dissector with monopolar cautery, electrosurgery probe, Veress needle	
De Sousa Martins *et al*.^44^	2018	Portugal	2014	PS	ND: GIA stapler	Compare safety and economic outcomes of new disposable and reprocessed disposable bipolar staplers and scissors for GLP
					ND: Harmonic	
					RD: GIA stapler	
					RD: Harmonic	
Dos Santos *et al*.^45^	2008	Brazil	NA	PS	RD: 5 mm, 11 mm and 12 mm trocars	Evaluate decontamination of reprocessed disposable trocars for LC
Gärtner *et al*.^46^	2008	Germany	2004–2005	PS	ND: ultrasonic scissors	Compare reliability of new disposable and reprocessed ultrasonic scissors for GLP
					RD: ultrasonic scissors	
Gundogdu *et al*.^47^	1998	Turkey	NA	PS	ND: trocars	Compare decontamination of new disposable and reprocessed disposable trocars for LC
					RD: trocars	
Jokar *et al*.^48^	2022	Iran	2018–2020	PS	ND: 5 mm and 11 mm trocars	Compare decontamination (in terms of PSI) of new disposable and reprocessed disposable trocars for LC
					RD: 5 mm and 11 mm trocars	
Lester *et al*.^49^	2010	USA	NA	AS	ND: Harmonic scalpel	Compare safety and performance of new disposable and reprocessed disposable Harmonic for GLP
					RD: Harmonic scalpel	
Mihanovic *et al*.^50^	2021	Croatia	2019–2020	RCT	ND: Harmonic scalpel	Compare safety of new disposable and reprocessed disposable Harmonic for LA
					RD: Harmonic scalpel	
Mues *et al*.^51^	2010	USA	NA	IVS and AS	ND: 5 mm bladed trocars and 5 mm bladeless trocar	Compare performance of new disposable and reprocessed disposable trocars for GLP
					RD: 5 mm bladed trocars and 5 mm bladeless trocar	
Weld *et al*.^52^	2005	USA	NA	PS	ND: Ethicon Shears LCS-C5	Compare safety and performance of new disposable and reprocessed disposable harmonic scalpels for GLP
					RD: Ethicon Shears LCS-C5	

AS, animal study; DLIs, disposable laparoscopic instruments; GLP, general laparoscopic practice; IVS, in-vitro study; LA, laparoscopic appendectomy; LC, laparoscopic cholecystectomy; LCO, laparoscopic colectomy; ND, new disposable; PS, prospective study; PSI, port site infection; R, Reusable; RCT, randomized controlled trial; RD, reprocessed disposable.

Within the 38 studies comparing disposable and reusable instruments, the majority (*n*=26) focused on general surgery^15,27–51^, with 30% (*n*=8) specifically examining the laparoscopic cholecystectomy (LC)^15,27,29,31,32,41,42,51^. The remaining 14 out of 26 studies broadly addressed instruments for endoscopic surgery or materials for multiple procedures. The other 12 studies comparing disposables and reusables included studies on bariatric^52,53^ (*n*=2), gynecological^14,54–59^ (*n*=7), pediatric^60,61^ (*n*=2), and thoracic surgery^62^ (*n*=1). In the bariatric studies, one focused on Roux-en-Y Gastric Bypass^52^, while the other explored instrument usage across various procedures^53^. Gynecological studies predominantly emphasized laparoscopic hysterectomy (*n*=3)^14,54,55^, with two studies also investigating the hysterectomy^56,58^. Pediatric studies focused on the appendectomy (*n*=2)^60,61^, and the article on thoracic surgery investigated instruments for sympathectomy^62^.

The 15 studies comparing new and reprocessed disposables all focused on general surgery and primarily addressing the cholecystectomy, colectomy, and appendectomy (see Table 1B)^63–77^. However, the type of procedure was unspecified in majority of the studies (*n*=9)^64–66,68,69,71,74,76,77^.

### Environmental impact

Eight studies investigated the environmental impact of instruments in endoscopic surgery^14,15,27,31,38,50,52,61^. These studies, encompassing a range of surgical procedures, are summarized in Table B1 (Supplemental Digital Content 4, http://links.lww.com/JS9/D563)^14,15,27,31,38,50,52,61^. Seven favored reusable instruments, while one lacked comparable outcome measures for differentiation.

Of the eight studies, five conducted a Life-Cycle Assessments (LCA) quantifying environmental impacts in terms of climate change (CO_2_ equivalent [CO_2_eq]), ecosystem damage (potentially disappeared fraction of species on the same surface per year [PDF×m×2yr]), human health damage (disability-adjusted life years [DALYs]), and resource depletion (US$)^14,15,31,38,50^. The remaining three studies provided outcomes such as total material requirements (kilograms) or waste production (kilograms) without specifying the analytical method^27,52,61^.

Meissner *et al*. observed a 300 g reduction in total material requirements and a 48.70–50% decrease in total waste production per sleeve gastrectomy or Roux-en-Y gastric bypass when utilizing reusable staplers instead of disposable ones^53^.The CO_2_eq from the lithium content in the multiuse stapler was 99.7% lower compared to the disposable alternatives, determined by multiplying the lithium mass by the emission factor^52^.

Among the studies on general surgery, Adler *et al*.^27^ compared the waste production of disposable trocars, scissors, and Veress cannula to all other instruments used in a LC. Single-use instruments generated 1·16 kg of household waste, 0·56 kg of cardboard waste, and 1·47 kg of plastic waste^27^. Boberg *et al*.^31^ conducted an LCA, demonstrating a median difference of 446 kg of CO_2_eq, 79 PDF×m2×yr, 2·4e-4 DALY/person/year, and 5160 megajoule (MJ) of resources between disposable and reusable trocars for one LC. Disposable stainless-steel instruments had a CO_2_eq. Forty percent greater than their plastic counterparts and 94% higher than reusable stainless-steel scissors^38^. Rizan and Bhutta^15^ study on LC highlighted the environmental superiority of reusable trocars, scissors, and clip appliers with cartridges over disposables, considering climate change, ecosystem quality, human health, and resources. In another study, the same author revealed higher CO_2_eq per LC for disposable instruments, such as 0·65 kg and 1·08 kg for 5 mm and 12 mm trocars, contrasting with reusable counterparts at 0·25 kg for a laparoscope, 0.13–0.23 kg for forceps, and 0.17 kg for a retractor^50^.

Thiel *et al*.^14^ reported a reduction of 110 kg CO_2_eq per hysterectomy when using reusable instruments compared to disposable ones. Furthermore, in a single-incision pediatric endoscopic appendectomy, a disposable stapler produced 581 g metal, 12 g paper, and 381 g plastics, while a reusable clip applier generated only 9 g paper and 9 g plastics^61^.

### Costs

Cost comparisons of disposable and reusable instruments were conducted in 20 studies, with unanimous findings indicating an economic advantage favoring reusable instruments^15,27,29,31–35,38,41,43,51,53–57,59–61^. The factors considered in the cost-estimation varied significantly among the studies, ranging from only the purchasing costs to a much broader scope, including processing costs, costs for instrument repair and replacement, and costs of disposal (see Tables 2A, 2B).

**Table 2A:** Cost outcomes of studies comparing disposables with reusables (*n*=20).

Author	Year	Procedure	Cost reduction reusable vs disposable	Factors included in cost-estimation			
				Purchase	Associated costs^a^	Processing costs^b^	Disposal^c^
Bariatric surgery (*n*=1)							
Yung *et al*.^2^	2010	LBS	29%	+	–	+	+
General surgery (*n*=12)							
Adler *et al*.^3^	2005	LC	95%	+	+	+	+
Apelgren *et al*.^5^	1993	LC	87%-91%	–	+	+	–
Boberg *et al*.^7^	2022	LC	42%	+	+	+	+
Demoulin *et al*.^8^	1996	LC	94%	+	+	+	+
DesCôteaux *et al*.^9^	1998	GLP	0·6%-79%	+	+	+	–
Eddie *et al*.^10^	1996	GLP	76%-87%	+	+	+	+
Fengler *et al*.^11^	1998	GLP	93%	+	+	–	–
Ibbotson *et al*.^14^	2013	NA	44%	+	+	+	+
MacFadyen *et al*.^15^	1994	LC	38%	+	+	+	+
Manatakis *et al*.^18^	2014	GLP	89%	+	+	+	+
Rizan *et al*.^25^	2022	LC	54%	+	+	+	+
Slater *et al*.^27^	2009	LC	84%	+	+	+	+
Gynecological surgery (*n*=5)							
Hasanov *et al*.^28^	2017	LH	57%	+	–	–	+
Holloran-Schwartz *et al*.^29^	2016	LH	95%	+	+	+	+
Martínez-Zamora *et al*.^30^	2009	LH & LM	95%	+	–	–	–
Ransom *et al*.^31^	1996	GL	98%	+	+	–	+
Schaer *et al*.^33^	1995	GL	87%	+	+	+	+
Pediatric surgery (*n*=2)							
Ebeid *et al*.^35^	2016	PLA	$300 (using reusables)	NA	NA	NA	NA
Graham *et al*.^36^	2019	SIPESA	$286–333 (using clips)	+	–	–	–

+ = included in cost-estimation; − = not included in cost-estimation.

^a^ Associated costs: instrument repair and replacement.

^b^ Processing costs: labor costs for materials management personnel to purchase, stock, store, and distribute instruments.

^c^ Disposal: waste disposal or cleaning and sterilization.

GL, gynecologic laparoscopy; GLP, general laparoscopic procedures; LBS, laparoscopic bariatric surgery; LC, laparoscopic cholecystectomy; LH, laparoscopic hysterectomy; LM, laparoscopic myomectomy; NA, not available; PLA, pediatric laparoscopic appendectomy; R, reusable; SIPESA, Single-Incision Pediatric Endosurgery Appendectomy; USD, US Dollars ($).

**Table 2B:** Cost outcomes of studies comparing new with reprocessed disposables (*n*=3).

Author	Year	Procedure	Cost reduction reusable vs disposable	Factors included in cost-estimation			
				Purchase	Associated costs^a^	Processing costs^b^	Disposal^c^
General surgery (*n*=3)							
Brady *et al*.^38^	2017	LCO	55%	+	+	+	+
Colak *et al*.^42^	2004	LC	56%	+	NA	+	NA
De Sousa Martins *et al*.^44^	2018	GLP	52%-57%	+	+	+	–

+ = included in cost-estimation; − = not included in cost-estimation.

^a^ Associated costs: instrument repair and replacement.

^b^ Processing costs: labor costs for materials management personnel to purchase, stock, store and distribute instruments.

^c^ Disposal: waste disposal or cleaning and sterilization.

GLP, general laparoscopic practice; LC, laparoscopic cholecystectomy; LCO, laparoscopic colectomy; NA, not available.

Yung *et al*.^53^ revealed a 29% price reduction in laparoscopic bariatric procedures by using reusable ultrasonic shears. Among the studies on general surgery (*n*=12) the cost decreases ranged for reusables from 38 to 95% (see Table 2A)^15,27,29,31–35,38,41,43,51^. Only in one study, comparing a limited-reusable set (17 reuses) to a reusable hook cautery (50 reuses), a minimal cost reduction of 0.6% was identified (see Table 2A)^33^.

Hasanov *et al*.^54^ reported a 50% cost reduction using reusable bipolar vessel-sealing devices compared to disposable Ligasure. Hollaran *et al*.^55^ found a potential cost decrease of 95% comparing Ligasure to bipolar forceps with monopolar scissors. Martinez-Zamora *et al*.^56^ estimated a cost of €907 for a disposable eclectic morcellator versus €45 for a reusable variant. Reusable 10 mm infraumbilical cannula usage resulted in a 98% cost reduction^57^. Schaer *et al*.^59^ identified 87% cost difference between single-use and reusable variants in laparoscopic gynecological surgery (see Table 2A).

In pediatric studies on appendectomy, reusable trocars resulted in $300 cost savings compared to disposables, while the use of a reusable clip applier resulted in $286–333 lower expenses to staplers or bipolar forceps^60,61^ (see Table 2A).

Examining the economic impact of reprocessed disposable instruments, all three studies favored reprocessed disposables over new ones. Brady *et al*.^63^ found a 55% cost reduction per laparoscopic colectomy for Ligasure reuse. Colak *et al*.^67^ reported a 56% reduction in expenditure for LC using reprocessed trocars, dissectors, curved scissors, jaws, graspers, hooks, and clips. Another study showed a 52–57% cost reduction for reprocessed GIA staplers and Harmonics^69^ (see Table 2B).

### Instrument performance

Sixteen studies assessed the performance of endoscopic surgical instruments in various surgical specialties (see Table B3.1-B3.2, Supplemental Digital Content 4, http://links.lww.com/JS9/D563)^33,37,39,40,42,44,46,49,53–58,61,62^. Due to a wide array of instruments and variables outcomes exhibited significant diversity. Seven studies demonstrated superior instrument performances for disposables, one favored reusables, and eight studies reported no significance difference between the two types (see Tables 3A, 3B).

**Table 3A:** Summary of environmental impact, costs, instrument performance and contamination risk of studies comparing disposables with reusables.

Author	Procedure	Year	Environmental impact	Costs	Instrument performance	Contamination risk
Bariatric surgery (*n*=2)						
Meissner *et al*.^1^	SG and RYGB	2023				
Yung *et al*.^2^	LBS	2010			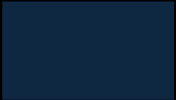	
General surgery (*n*=26)						
Adler *et al*.^3^	LC	2005	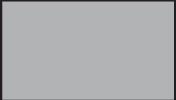			
Alfa *et al*.^4^	GLP	2003				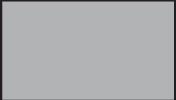
Apelgren *et al*.^5^	LC	1993				
Baxter *et al*.^6^	GLP	2006				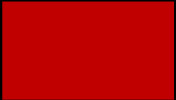
Boberg *et al*.^7^	LC	2022				
Demoulin *et al*.^8^	LC	1996				
DesCôteaux *et al*.^9^	GLP	1998			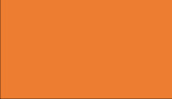	
Eddie *et al*.^10^	GLP	1996				
Fengler *et al*.^11^	GLP	1998				
Fengler *et al*.^12^	GLP	2000				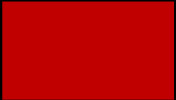
Harper *et al*.^13^	GLP	2009			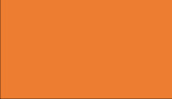	
Ibbotson *et al*.^14^	GLP	2013				
Kelty *et al*.^15^	GLP	2000			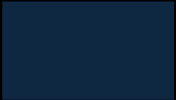	
Klar *et al*.^16^	GLP	2011				
MacFadyen *et al.* ^15^	LC	1994				
Mahmoud *et al*.^17^	LC	2019			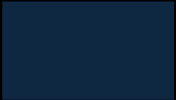	
Manatakis *et al*.^18^	GLP	2014				
Montero *et al*.^19^	GLP	2010			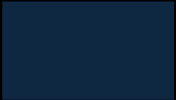	
Murdoch *et al*.^20^	GLP	2006				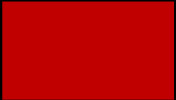
Nezhat *et al*.^21^	GLP	1991			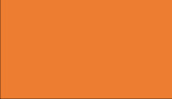	
Owusu *et al*.^22^	GLP	2022				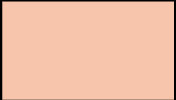
Raina *et al*.^23^	GLP	2018				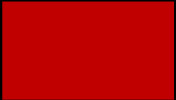
Richter *et al*.^24^	GLP	2006			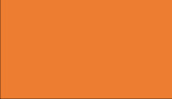	
Rizan *et al*.^25^	LC	2022				
Rizan *et al*.^26^	LC	2023				
Slater *et al*.^27^	LC	2009				
Gynecological surgery (*n*=7)						
Hasanov *et al*.^28^	LH	2017			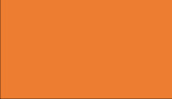	
Holloran-Schwartz *et al*.^29^	LH	2016			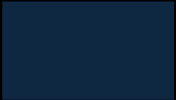	
Martínez-Zamora *et al*.^30^	LH & LM	2009			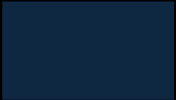	
Ransom *et al*.^31^	GL	1996			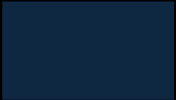	
Rothmund *et al*.^32^	LASH	2013			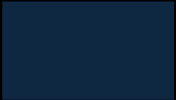	
Schaer *et al*.^33^	GL	1995				
Thiel *et al*.^34^	LH	2018				
Pediatric surgery (*n*=2)						
Ebeid *et al*.^35^	PLA	2016				
Graham *et al*.^36^	SIPESA	2019			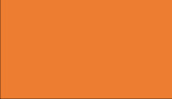	
Thoracic surgery (*n*=1)						
Weksler *et al*.^37^	ETS	2009			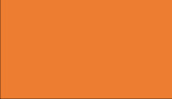	


 Favoring reusable instruments: The study indicates a difference in the investigated outcome, with results favoring reusable over disposable instruments.

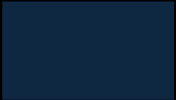
 Favoring disposable instruments: The study indicates a difference in the investigated outcome, with results favoring disposable over reusable instruments.

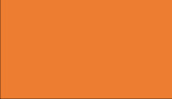
 No difference between disposable and reusable instruments: The study found no difference between disposable and reusable instruments in the investigated outcome.

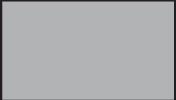
 Data missing: The study did not provide sufficient data for a comparison between disposable and reusable instruments.

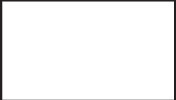
 Not investigate: The particular outcome was not included in the study’s scope.

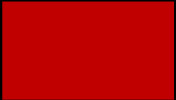
 Residual contamination: The study detected residual contamination on reusable instruments after cleaning and sterilization.

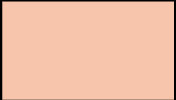
 No residual contamination: The study found no residual contamination on reusable instruments following cleaning and sterilization.

ETS, endoscopic thoracic sympathectomy; GL, gynecologic laparoscopy; GLP, general laparoscopic practice; LASH, laparoscopic supracervical hysterectomy; LBS, laparoscopic bariatric surgery; LC, laparoscopic cholecystectomy; LH, laparoscopic hysterectomy; LM, laparoscopic myomectomy; PLA, pediatric laparoscopic appendectomy; RYGB, Roux-en-Y Gastric Bypass; SG, sleeve gastrectomy; SIPESA, single-incision pediatric endosurgery appendectomy.

**Table 3B:** Summary of costs, instrument performance and contamination risk of the studies comparing new with reprocessed disposables.

Author	Procedure	Year	Costs	Instrument performance	Contamination risk
General surgery (*n*=15)					
Brady *et al*.^38^	LCO	2017		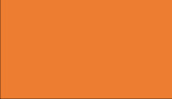	
Carungi *et al*.^39^	GLP	NA			
Chan *et al*.^40^	GLP	2000			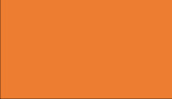
Chivukula *et al*.^41^	GLP	2020			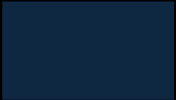
Colak *et al*.^42^	GLP	2004		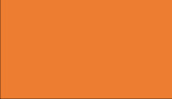	
De Lion Botero Couto Lopes *et al*.^43^	GLP	2011			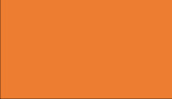
De Sousa Martins *et al*.^44^	GLP	2018		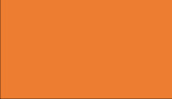	
Dos Santos *et al*.^45^	LC	2008			
Gärtner *et al*.^46^	GLP	2008		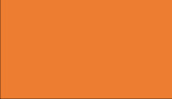	
Gundogdu *et al*.^47^	LC	1998			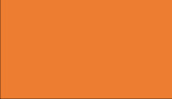
Jokar *et al*.^48^	LC	2022			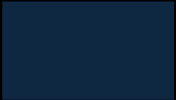
Lester *et al*.^49^	GLP	2010		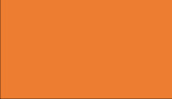	
Mihanovic *et al*.^50^	LA	2021		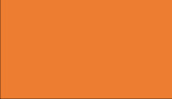	
Mues *et al*.^51^	GLP	2010		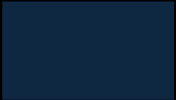	
Weld *et al*.^52^	GLP	2005		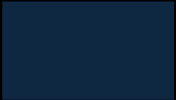	


 Favoring reprocessed disposable instruments: The study indicates a difference in the investigated outcome, with results favoring reprocessed over new disposable instruments.

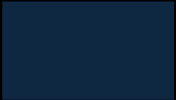
 Favoring new disposable instruments: The study indicates a difference in the investigated outcome, with results favoring new disposable instruments over reprocessed ones.

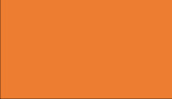
 No difference between new and reprocessed disposable instruments: The study found no difference between new and reprocessed disposables in the investigated outcome.

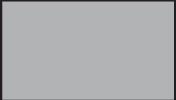
 Data missing The study did not provide sufficient data for a comparison between new and reprocessed disposables.

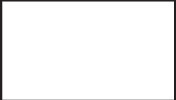
 Not investigated in study: The particular outcome was not included in the study’s scope.

GLP, general laparoscopic practice; LA, laparoscopic appendectomy; LC, laparoscopic cholecystectomy; LCO, laparoscopic colectomy.

Yung *et al*.^53^ found no significant differences in operating time and blood loss between disposable and reusable ultrasonic shears in laparoscopic bariatric surgery. Klar *et al*.^40^ reported a significant reduction in sealing time for reusable MarSeal compared to Ligasure. Complementing these findings, Mahmoud and El-Atar^42^ observed prolonged operating times and hospital stays with reusable electrosurgical energy dissectors versus harmonics in LC, while complications were consistent. By comparing the disposable ligasure to the reusable biclamp a statistically significant divergence was only revealed in tissue charring score (1.30 for ligasure vs. 1.64 for biclamp)^49^.

DesCôteaux reported comparable satisfaction ratings between limited-reusable scissors and hook cautery (13–17 times reuse) and their reusable (25–50 times reuse) counterparts^33^. Assessment of Direct Drive, Endopath, and Auto Suture Endo shears revealed similar cutting and ergonomics scores, with imperfection rates of 0.09, 1.18, and 1.09 per millimeter^37^. Montero *et al*.^44^ observed a significant difference in insulation failure between disposable L-hook (3%) and reusable instruments (19%).

Kelty and Super^39^ found a 2.04 pounds per square inch (PSI) lower force requirement for inserting disposable trocars in a reconstructed abdominal wall. Nezhat *et al*.^46^ identified no statistically significant differences in complications and failed insertions between disposable and reusable trocars.

In two gynecological studies, no significant differences were observed in operating time, blood loss, complications, and hospital stay between disposable Ligasure and reusable Marseal^54,55^. However, Holloran-Schwartz noted significantly shorter cutting times with Ligasure compared to bipolar forceps (8.4 vs. 14.6 min)^55^. Three studies favored disposable instruments, particularly for electric morcellators, where morcellating rate and piece length favored disposables, although reusable morcellating time was shorter^56–58^. Ransom *et al*.^57^ preferred disposable cannulas for lower bowel injury incidence. Comparing single-use Enseal with a bipolar device, a significantly shorter operating time and hospital stay were evident for the disposable alternative^58^.

In single-incision pediatric appendectomy, no significant differences were found in operating time, blood loss, hospital stay, and complications between disposable stapler and reusable clip appliers^61^. For thoracic sympathectomy, no disparities were observed in hospital stay and complications between disposable ultrasonic scalpel and reusable monopolar hook cautery^62^.

Nine studies evaluated new and reprocessed disposable instruments (see Table B3.2, Supplemental Digital Content 4, http://links.lww.com/JS9/D563)^63,64,67,69,71,74–77^. Three studies showed significant advantages for new over reprocessed disposables^64,76,77^. Carungi found a significant difference in the number of 300 successive activations between new and reprocessed Harmonic instruments, with new instruments achieving 22 out of 30 activations, compared to 30 out of 30 for reprocessed instruments; the latter underwent disassembly and revision of all components^64^. Mues *et al*.^76^ reported a lower air leak rate for new trocars compared to reprocessed ones (78.5–89.3 ml/min versus 161.2–169.0 ml/min). A significantly lower number of abnormalities were observed in new Ethicon Shears following visual inspection^77^. The six studies without significant differences explored various instruments, including new versus reprocessed Harmonics^69,74,75^, LigaSure^63^, instruments for LC^67^, and ultrasonic scissors^71^ (see Table B3.2, Supplemental Digital Content 4, http://links.lww.com/JS9/D563).

### Contamination risk

No studies were found comparing the contamination risk between disposable and reusable instruments. Six studies investigated the infection risk associated with reusable instruments (see Table B4.1, Supplemental Digital Content 4, http://links.lww.com/JS9/D563)^28,30,36,45,47,48^. Residues were detected in four studies, while one study reported the absence of residues, and another study lacked clear data (see Table B4.1).

Five investigations assessed the contamination risk through residual quantities or bacterial proliferation^28,30,36,45,47^. Alfa *et al*.^28^ utilized an artificial test soil to simulate infection risk, achieving a 99.5–100% reduction in protein, carbohydrate, and hemoglobin volumes after cleaning cleaner. Four studies analyzed residual proteins or living bacterial contaminants on instruments from actual surgeries. Baxter *et al*.^30^ found median residual proteins ranging from 163 µg to 267 µg on instruments in surgical trays. Additionally, Fengler *et al*.^36^ reported that 25% of laparoscopic tray instruments still contained proteins, with 12.5% showing positive hemoglobin stick test results. Murdoch *et al*.^45^ identified high percentages of residual proteins in specific instruments, including retractors (43.47%), scissors (28.57%), forceps (37.93%), gags (50%), Birkett (66·66%), and dissectors (40%). In another study, positive living bacterial isolates for Bacillus cereus and Citrobacter freundii were only found on Kocher forceps, mayo scissors, and Deaver retractor^47^. No bacterial residues were detected on dissecting forceps, Rampley sponge-holding forceps, curved mosquito artery forceps, Metzenbaum scissors, and Langenberg retractor after autoclavation^47^. However, none of these studies addressed potential patient consequences, such as disease transmission or wound infection.

Only one study, conducted by Raina *et al*.^48^, assessed patient outcomes related to infections. Among 112 patients undergoing laparoscopic procedures with reusable instruments, 3.57% experienced superficial port-site infections, and 1.78% had deep infections^48^.

Six studies investigated the contamination risk in reprocessed disposables (see Table B4.2, Supplemental Digital Content 4, http://links.lww.com/JS9/D563)^65,66,68,70,72,73^. Three studies found no difference between new and reprocessed disposables, two favored new disposables, and one reprocessed disposables. Five studies primarily examined positive cultures or residues without evaluating clinical consequences^65,66,68,70,72^. Three studies used instruments from patient care^66,70,72^, in one study instruments were infected with polio and herpes simplex virus type 1 (HSV) infections from preinfected horse blood^65^, and in another study, instruments were contaminated with specific bacterial strains^68^. Clinical implications were not assessed in these studies.

Chivukula’s comparative analysis of new and reprocessed Ligasure instruments uncovered residual contamination in the latter: 52% visually, 100% microscopically, 92% through scanning electron microscopy (SEM), and 50% via hemoglobin detection^66^. Dos Santos found no positive cultures in 28 reprocessed trocars after sterilization^70^. Gundogdu *et al*.^72^ reported a 3.33% positive culture rate for reprocessed trocars, statistically comparable to new ones. Chan *et al*.^65^ noted contamination in two out of four reprocessed trocar cultures with polio, while none tested positive for HSV. Instruments contaminated with Geobacillus stearothermophilus and Bacillus atrophaeus showed no residual contamination post-cleaning^68^.

A study examining port site infections as a clinical outcome found a significant difference between new and reprocessed trocars (1.9 vs. 8.5%, *P*=0.002, 3–14 days postsurgery)^73^. Notably, the study lacks details on confounding variables and specifics of the cleaning process^73^.

### Risk of bias

The five LCAs showed varied quality, completeness, and risk of bias, with critical appraisal scores ranging from 66 to 84%, indicating a moderate to low risk of bias (see Table S1, Supplemental Digital Content 5, http://links.lww.com/JS9/D565)^14,15,31,38,50^. Among randomized studies, two had a high risk of bias, and three raised some concerns (see Table S2, Supplemental Digital Content 5, http://links.lww.com/JS9/D565)^54–56,58,75^. Nonrandomized studies exhibited serious risk in 14 cases^27,29,33,35,36,39,43,48,51,57,59,60,70–73,77^ and moderate risk in 20^28,30,32,34,41,42,44–47,52,53,61–69,76^ (see Table S3, Supplemental Digital Content 5, http://links.lww.com/JS9/D565). The SYRCLE risk of bias tool for animal studies highlighted inadequately described experimental details, leading to ‘unclear risk of bias’ in many aspects^37,40,49,74^. Studies generally lacked sufficient reporting on critical elements, such as randomization, blinding, and power analysis/sample size calculation (see Table S4, Supplemental Digital Content 5, http://links.lww.com/JS9/D565).

## Discussion

Integrating planetary health principles into the choice of medical instrumentation requires a novel approach, something not addressed in current literature or practice. With instrumentation in disposable and reusable forms, decision-making can be complicated, with factors such as costs, performance, and contamination risk, influencing this choice. Endoscopic surgery, favored for its technical and clinical benefits, raises concerns due to the waste production^13–15^. This review evaluated the different outcomes for disposable and reusable instruments or new versus reprocessed disposables in endoscopic surgery.

This review analyzed 53 studies comparing disposable, reusable, or reprocessed instruments. Reusables demonstrated ecological advantages, with cost savings for both reusables and reprocessed disposables. Instrument performance was comparable between disposables and reusables. While no study addressed contamination risk for disposables, six studies on reusables and reprocessed disposables revealed residues after cleaning and sterilization, although clinical implications were unclear.

A common argument for using disposables is patient safety, particularly an assumed lower infection risk. But despite studies detecting residual proteins or bacteria on reusables, their clinical impact remains unclear if not absent^28,30,36,45,47,48^. Reusable instruments in open surgery, like forceps and wound retractors, are used without increased infection risk, suggesting concerns may be overstated. Since reusables in open surgery directly contact tissues, their safe use may also translate to endoscopic instruments. While endoscopic instruments are often rigorously tested, instruments used for skin closure during the same procedure do not raise contamination concerns. Given the absence of evidence supporting a higher infection risk associated with reusables compared to disposables, the use of disposables should be reconsidered.

The review also shows cost reductions for reusables or reprocessed disposables compared to new disposable instruments, varying from 0·6 to 98%. Contrary to the belief of disposables becoming cheaper over time, our analysis spanning from 1993 to 2022 shows that reusables consistently maintain an economic advantage, contributing to a more nuanced discussion on instrument preferences.

Despite the environmental benefits of reusables, disposables still dominate endoscopic surgery. Disposables contribute significantly to emissions in various medical fields, including ophthalmology, where over 30% of emissions come from equipment^78^. Similarly, in bladder and carpal tunnel surgery, disposables contribute heavily to emissions^79,80^. In delivery rooms, reusables produce lower emissions after only three uses, while 25% of emissions in renal services stem from disposables^81,82^. Across specialties, reusables consistently show environmental benefits.

Besides choosing disposables or reusables, a third option involves reprocessing disposables. While this review indicates that reprocessed disposables result in cost savings, their potential for reducing environmental impact may not surpass the climate-friendly optimization achievable with reusable instruments, especially when considering the 10R framework of a circular economy^83^. The 10R’s, structured in a hierarchy based on their emissions-minimizing effectiveness, include refuse, reduce, rethink, reuse, repair, refurbish, remanufacture, repurpose, recycle, and recover measures^83^. Reprocessing disposables, with their limited lifespan, may be less long-term beneficial than reusables. However, regulatory challenges hinder the adoption of a circular economy in healthcare and adapting regulations is essential to support climate-friendly medical practices.

Technological advancements could offer a promising path toward innovative and sustainable surgery^84^. While studies on new instruments were not included, endoscopic surgery is evolving with initiatives focused on combining environmental sustainability with positive patient outcomes^84^. However, as most of these initiatives are not yet ready for clinical use, they should complement, rather than replace, the 10R model.

Currently, the ongoing debate is usually about the balance between costs and environmental impact on one hand, and the idea that patient outcomes may be compromised at the other. However, the latter is not supported by evidence. Ethical considerations become crucial when choosing sustainable alternatives^85,86^. We must assess whether evacuating all potential patient risk factors, if proven, would inflict more harm on individual patients than the healthcare’s impact on the planet. Is clinical practice truly beneficial if we choose less sustainable options posing threats to the environment and health of present and future generations? A framework and behavioral change are necessary to weigh patient-related outcomes, costs, and environmental impact, establishing a best practice aligned with planetary health^87–89^.

This study acknowledges limitations, mainly the low to moderate quality of included studies, mostly retrospective in design. Heterogeneity in populations, interventions, and outcomes prevented a meta-analysis. The intrinsic variability in study designs and methodologies across the studies challenged the overall robustness of the results.

In conclusion, this review advocates using reusable endoscopic surgical instruments, emphasizing their environmental and cost benefits. Integrating considerations of planetary health is crucial, highlighting the environmental benefits of reusables in reducing pollution. Balancing outcomes is essential for the patient and planetary benefits. Limited and controversial evidence regarding contamination risk emphasizes the need for further research. The widely held belief that disposables effectively prevent cross-contamination requires reassessment, with greater consideration given to their impact on the planet and the resulting effects on public health.

## Ethical approval

Not applicable.

## Consent

Not applicable.

## Source of funding

All authors were not precluded from accessing data in the study and accept responsibility to submit for publication. All authors have no disclosures on funding recourses. No author was paid to write this article by a company or other agency.

Prof. dr. Nicole Bouvy was granted by NWO (Dutch Research Council for the CAREFREE-consortium.

## Author contribution

M.E., M.M., L.W., and N.B.: had full access to all data in the study and take responsibility for the integrity of the data and accuracy of data analysis; M.E., M.M., and N.B.: study concept and design; M.E., M.M., L.W., and N.B.: drafting of the manuscript; P.R., T.S., L.B., and M.K.: study supervision. All authors contributed in acquisition, analysis, or interpretation of the data, and reviewing the manuscript.

## Conflicts of interest disclosure

All authors were not precluded from accessing data in the study and accept responsibility to submit for publication. All authors have no disclosures on funding recourses. No author was paid to write this article by a company or other agency.

## Research registration unique identifying number (UIN)

PROSPERO under the registration number CRD42023420354.

## Guarantor

Nicole Bouvy and Myrthe Eussen.

## Data availability statement

Data sharing is not applicable for this systematic review as no new data were generated or analyzed during the project. All results presented in this manuscript and supplementary materials are directly based on data from the original articles, which are cited in the references. For further information, please contact the corresponding author.

## Provenance and peer review

Not commissioned, externally peer-reviewed.

## Financial support and sponsorship

Not applicable.

## Presentation

Not applicable.

## Supplementary Material

SUPPLEMENTARY MATERIAL
