# Supplementary material for: Beyond single-use: a systematic review of environmental, economic, and clinical impacts of endoscopic surgical instrumentation
Source: Int J Surg. 2024 Nov 18;110(12):8136–50. doi: 10.1097/JS9.0000000000002141 (PMC11634193; doi:10.1097/JS9.0000000000002141)
Supplement: SUPPLEMENTARY MATERIAL [file js9-110-8136-s003.docx]

**Appendix A: Search strategy for the different literarture databases on articles to include**

| ***Database*** | ***Search syntax*** |
| --- | --- |
| ***General part*** | |
| Pubmed | ((((((((((((((((("Surgical Equipment"[Mesh:NoExp]) OR ("Endoscopes"[Mesh:NoExp])) OR ("Laparoscopes"[Mesh])) OR ("Mediastinoscopes"[Mesh])) OR ("Thoracoscopes"[Mesh])) OR ("Endoscopy"[Mesh:NoExp])) OR ("Laparoscopy"[Mesh])) OR ("Mediastinoscopy"[Mesh])) OR ("Natural Orifice Endoscopic Surgery"[Mesh])) OR ("Thoracoscopy"[Mesh])) OR ("Video-Assisted Surgery"[Mesh])) OR (instrument*[Tiab])) OR (endoscop*[Tiab])) OR (laparoscop*[Tiab])) OR (mediastinoscop*[Tiab])) OR (thoracoscop*[Tiab])) AND ((((("Disposable Equipment"[Mesh]) OR (dispos*[Tiab])) OR (one-time[Tiab])) OR (single-use[Tiab])) OR (((("Equipment Reuse"[Mesh]) OR ("Recycling"[Mesh])) OR (reus*[Tiab])) OR (recycl*[Tiab])))) |
| Embase | surgical equipment/ OR endoscopic surgery/ or laparoscopy/ or natural orifice transluminal endoscopic surgery/ or transanal endoscopic surgery/ or video assisted surgery/ OR endoscopy/ or mediastinoscopy/ or thoracoscopy/ OR endoscope/ or digestive endoscope/ or laparoscope/ or robotic endoscope/ or thorax endoscope/ OR exp laparoscopic surgery/ or exp laparoscopic surgical instrument/ OR "instrument*".ab,kw,ti. OR "endoscop*".ab,kw,ti. OR "laparoscop*".ab,kw,ti.OR "mediastinoscop*".ab,kw,ti.OR "thoracoscop*".ab,kw,ti.AND exp disposable equipment/ OR "dispos*".ab,kw,ti. OR one-time.ab,kw,ti. OR single-use.ab,kw,ti.AND exp recycling/ OR "reus*".ab,kw,ti. OR "recycl*".ab,kw,ti. |
| Web of Science | ((TS=(instrument*)) OR TS=(endoscop*)) OR TS=(laparoscop*) AND ((TS=(dispos*)) OR TS=(one-time)) OR TS=(single-use) AND (TS=(reus*)) OR TS=(recycl*) |
| The Cochrane Library | [Surgical Equipment] this term only OR [Surgical Instruments] this term only OR [Endoscopes] this term only OR [Laparoscopes] explode all trees OR  [Mediastinoscopes] explode all trees OR [Thoracoscopes] explode all trees OR [Endoscopy] this term only OR [Laparoscopy] explode all trees OR [Mediastinoscopy] explode all trees OR [Thoracoscopy] explode all trees OR [Minimally Invasive Surgical Procedures] explode all trees instrument* OR endoscop* OR laparoscop* OR mediastinoscop* OR thoracoscop* AND [Disposable Equipment] explode all trees OR dispos* OR one-time OR single-use AND [Equipment Reuse] explode all trees OR reus* OR recycl* |
| ***Outcome: environmental impact*** | |
| Pubmed | AND ((((((((((((((((("Environment"[Mesh]) OR ("Climate Change"[Mesh])) OR ("Carbon Dioxide"[Mesh])) OR ("Environmental Pollutants"[Mesh])) OR ("Waste Management"[Mesh])) OR (sustainab*[Tiab])) OR (greenhouse*[Tiab])) OR (environment*[Tiab])) OR (global warming[Tiab])) OR (climat*[Tiab])) OR (carbon[Tiab])) OR (footprint*[Tiab])) OR (pollution*[Tiab])) OR (pollutant*[Tiab])) OR (life cycle assessment*[Tiab])) OR (life cycle analysis[Tiab])) OR (LCA[Tiab])) |
| Embase | AND exp climate change/ OR exp environmental footprint/ OR exp environmental impact/ OR environmental sustainability/ OR global climate/ OR carbon dioxide/ OR exp air pollution/ OR exp life cycle assessment/ OR exp environmental impact assessment/ OR greenhouse gas/ OR "sustainab*".ab,kw,ti. OR "greenhouse*".ab,kw,ti. OR "environment*".ab,kw,ti. OR global warming.ab,kw,ti. OR "climat*".ab,kw,ti. OR carbon.ab,kw,ti. OR "footprint*".ab,kw,ti. OR "pollution*".ab,kw,ti. OR "pollutant*".ab,kw,ti. OR "life cycle assessment*".ab,kw,ti. OR life cycle analysis.ab,kw,ti. OR LCA.ab,kw,ti. |
| Web of Science | (((((((((TS=(sustainab*)) OR TS=(greenhouse*)) OR TS=(environment*)) OR TS=(global warming)) OR TS=(climat*)) OR TS=(carbon)) OR TS=(footprint*)) OR TS=(pollutant*)) OR TS=(life cycle assessment*)) OR TS=(life cycle analysis) |
| The Cochrane Library | AND [Environment] this term only OR [Climate Change] explode all trees OR [Greenhouse Effect] explode all trees OR [Carbon Dioxide] this term only  OR [Environmental Pollutants] explode all trees OR [Waste Management] explode all trees OR sustainab* OR greenhouse* OR environment* OR global warming OR climat* OR carbon footprint* OR pollution* OR pollutant* OR life cycle assessment* OR life cycle analysis OR LCA |
| ***Outcome: costs*** | |
| Pubmed | AND (((("Disposable Equipment"[Mesh]) OR (dispos*[Tiab])) OR (one-time[Tiab])) OR (single-use[Tiab]))) AND (((("Equipment Reuse"[Mesh]) OR ("Recycling"[Mesh])) OR (reus*[Tiab])) OR (recycl*[Tiab]))) AND ((((((("Economics"[Mesh:NoExp]) OR ("economics"[Subheading])) OR ("Costs and Cost Analysis"[Mesh])) OR ("Economics, Hospital"[Mesh])) OR ("Economics, Medical"[Mesh])) OR (cost*[Tiab])) OR (economic*[Tiab])) |
| Embase | AND "cost"/ OR economics/ OR exp health economics/ OR exp economic evaluation/ OR "cost*".ab,kw,ti. OR "economic*".ab,kw,ti. |
| Web of Science | (TS=(cost*)) OR TS=(economic*) |
| The Cochrane Library | AND [Economics] this term only OR [Costs and Cost Analysis] explode all trees OR [Economics, Hospital] explode all trees OR [Economics, Medical] explode all trees OR cost* OR economic* |
| ***Outcome: instrument performance*** | |
| Pubmed | AND ((((((((((((((((("Equipment Failure"[Mesh:NoExp]) OR ("Equipment Failure Analysis"[Mesh])) OR ("Equipment Safety"[Mesh])) OR ("Ergonomics"[Mesh])) OR ("Materials Testing"[Mesh])) OR (ergonom*[Tiab])) OR (insulation[Tiab])) OR (dysfunction[Tiab])) OR (disfunction[Tiab])) OR (malfunction[Tiab])) OR (fail*[Tiab])) OR (quality[Tiab])) OR (safe*[Tiab])) OR (reliab*[Tiab])) OR (function*[Tiab])) OR (efficacy[Tiab])) OR (performance[Tiab)) |
| Embase | AND exp device failure/ OR exp device safety/ OR patient safety/ OR ergonomics/ OR mechanics/ OR "ergonom*".ab,kw,ti. OR dysfunction.ab,kw,ti. OR disfunction.ab,kw,ti. OR malfunction.ab,kw,ti. OR "fail*".ab,kw,ti. OR quality.ab,kw,ti. OR "safe*".ab,kw,ti. OR "reliab*".ab,kw,ti. OR "function*".ab,kw,ti. OR efficacy.ab,kw,ti. OR performance.ab,kw,ti. |
| Web of Science | ((((((((((TS=(ergonom*)) OR TS=( insulation)) OR TS=(dysfunction)) OR TS=(disfunction)) OR TS=(malfunction)) OR TS=(fail*)) OR TS=(quality)) OR TS=(safe*)) OR TS=(reliab*)) OR TS=(function*)) OR TS=(efficacy)) OR TS=(performance) |
| The Cochrane Library | AND [Equipment Failure] explode all trees OR [Equipment Safety] explode all trees OR [Ergometry] explode all trees OR [Ergonomics] this term only OR ergonom* OR insulation OR dysfunction OR disfunction OR malfunction OR fail* OR quality OR safe* OR reliab* OR function* OR efficacy OR performance |
| ***Outcome: contamination risk*** | |
| Pubmed | AND (((((((((((((((((((((((((((((((((((((((((("Infection Control"[Mesh]) OR ("Medical Waste"[Mesh])) OR ("Disease Transmission, Infectious"[Mesh])) OR ("Infections"[Mesh])) OR ("Iatrogenic Disease"[Mesh])) OR ("Decontamination"[Mesh])) OR ("Equipment Contamination"[Mesh])) OR ("Quality Control"[Mesh])) OR ("Organisms Category"[Majr])) OR ("Microbiology"[Mesh])) OR ("Anti-Infective Agents"[Mesh])) OR ("Surface-Active Agents"[Mesh])) OR ("Disease Outbreaks"[Mesh])) OR (infection*[Tiab])) OR (cross-infection*[Tiab])) OR (crossinfection*[Tiab])) OR (transmission[Tiab])) OR (contamination[Tiab])) OR (microorganism*[Tiab])) OR (microbiology*[Tiab])) OR (bacter*[Tiab])) OR (virus[Tiab])) OR (viral[Tiab])) OR (fung*[Tiab])) OR (parasit*[Tiab])) OR (steril*[Tiab])) OR (autoclave[Tiab])) OR (clean*[Tiab])) OR (disinfect*[Tiab])) OR (decontamin*[Tiab])) OR (detergent*[Tiab])) OR (antiinfective[Tiab])) OR (anti-infective[Tiab])) OR (antibacter*[Tiab])) OR (anti-bacter*[Tiab])) OR (anti-vir*[Tiab])) OR (antivir*[Tiab])) OR (antifug*[Tiab])) OR (anti-fug*[Tiab])) OR (antiparasitic[Tiab])) OR (anti-parasitic[Tiab])) OR (iatrogen*[Tiab)) |
| Embase | AND infection/ OR device infection/ OR infection control/ OR exp asepsis/ OR exp sterilizer/ OR exp antiinfective agent/ OR exp microbial contamination/ OR exp medical device contamination/ OR exp disease transmission/ OR iatrogenic disease/ OR infection risk/ OR exp iatrogenic Creutzfeldt Jakob disease/ OR infection prevention/ OR "infection*".ab,kw,ti. OR "cross-infection*".ab,kw,ti. OR "crossinfection*".ab,kw,ti. OR transmission.ab,kw,ti. OR contamination.ab,kw,ti. OR "microorganism*".ab,kw,ti. OR "microbiology*".ab,kw,ti. OR "bacter*".ab,kw,ti. OR virus.ab,kw,ti. OR viral.ab,kw,ti. OR "fung*".ab,kw,ti. OR "parasit*".ab,kw,ti. OR "steril*".ab,kw,ti. OR autoclave.ab,kw,ti. OR "clean*".ab,kw,ti. OR "disinfect*".ab,kw,ti. OR "decontamin*".ab,kw,ti. OR antiinfective.ab,kw,ti. OR anti-infective.ab,kw,ti. OR "antibacter*".ab,kw,ti. OR "anti-bacter*".ab,kw,ti. OR "anti-vir*".ab,kw,ti. OR "antivir*".ab,kw,ti. OR "antifug*".ab,kw,ti. OR "anti-fug*".ab,kw,ti. OR antiparasitic.ab,kw,ti. OR anti-parasitic.ab,kw,ti. OR "iatrogen*".ab,kw,ti. OR "detergent*".ab,kf,ti. |
| Web of Science | ((((((((((((((((((((((((((((TS=(infection*)) OR TS=(cross-infection*)) OR TS=(crossinfection*)) OR TS=(transmission)) OR TS=(contamination)) OR TS=(microorganism*)) OR TS=(microbiology*)) OR TS=(bacter*)) OR TS=(virus)) OR TS=(viral)) OR TS=(fung*)) OR TS=(parasit*)) OR TS=(steril*)) OR TS=(autoclave)) OR TS=(clean*)) OR TS=(disinfect*)) OR TS=(decontamin*)) OR TS=(detergent*)) OR TS=(antiinfective)) OR TS=(anti-infective)) OR TS=(antibacter*)) OR TS=(anti-bacter*)) OR TS=(anti-vir*)) OR TS=(antivir*)) OR TS=(antifug*)) OR TS=(anti-fug*)) OR TS=(antiparasitic)) OR TS=(anti-parasitic)) OR TS=(iatrogen*) |
| The Cochrane Library | AND [Infections] this term only OR [Cross Infection] explode all trees OR [Disease Transmission, Infectious] explode all trees OR [Equipment Contamination] explode all trees OR [Decontamination] explode all trees OR [Communicable Disease Control] explode all trees OR [Anti-Infective Agents] explode all trees OR infection* OR cross-infection* OR crossinfection* OR transmission OR contamination OR microorganism* OR microbiology* OR bacter* OR virus OR viral OR fung* OR parasit* OR steril* OR autoclave OR clean* OR disinfect* OR decontamin* OR detergent* OR antiinfective OR anti-infective OR antibacter* OR anti-bacter* OR anti-vir* OR antivir* OR antifug* OR anti-fug* OR antiparasitic OR anti-parasitic OR iatrogen* |
