# Supplementary material for: Beyond single-use: a systematic review of environmental, economic, and clinical impacts of endoscopic surgical instrumentation
Source: Int J Surg. 2024 Nov 18;110(12):8136–50. doi: 10.1097/JS9.0000000000002141 (PMC11634193; doi:10.1097/JS9.0000000000002141)
Supplement: SUPPLEMENTARY MATERIAL [file js9-110-8136-s004.docx]

**Appendix B: summary of different outcomes**

**Table B1: Environmental outcome of studies comparing disposables with reusables (n=8)**

| ***Author*** | ***Procedure*** | ***Included instruments*** | ***No. of reuses*** | ***Type of LCA (guideline)*** | ***Approach*** | ***Outcome*** |
| --- | --- | --- | --- | --- | --- | --- |
| *Bariatric surgery (n=1)* | | | | | | |
| Meissner et al. ^43^ | SG & RYGB | D: stapler | 1 | NA | NA | Per SG:   - TMR^III^: 0.33kg - Total waste production: 1.62kg   Per RYGB:   - TMR^III^: 0.33kg - Total waste production: 1.54kg   Climate change (CO_2_eq) of lithium content: 0.006kg |
|  |  | R: stapler | 50 | NA | NA | Per SG:   - TMR^III^: 0.03kg - Total waste production: 0.81kg   Per RYGB:   - TMR^III^: 0.03kg - Total waste production: 0.79kg   Climate change (CO_2_eq) of lithium content: 18e^-6^kg |
| *General surgery (n=5)* | | | | | | |
| Adler et al. ^18^ | LC | D: trocars, scissors and Veress cannula | 1 | NA | NA | Waste production per case (kg)   - Household: 1.16 - Cardboard: 0.56 - Plastics: 1.47 |
|  |  | R: all other instruments | 255 |  |  | Use of recourses per case for cleaning and sterilization   - Water: 1.46m^3^ - Energy: 20.58kWh - Cleaning agents: 0.3L - Steam: 0.018kg - Compressed air: 0.13m^3^ |
| Boberg et al. ^22^ | LC | D: trocars | 1 | Hybrid  (ISO 14044) | Cradle to grave | Median difference:   - Climate change (CO_2_eq): 446kg - Ecosystem quality^I^ (PDF*m2*yr): 79 - Human health^II^ (DALY/person/yr): 2.4e^-4^ - Resources (MJ): 5160 |
|  |  | R: trocars | 100-500 |  |  |  |
| Ibbotson et al. ^29^ | NA | D: plastic scissors | 1 | EIO  (ISO 14040) | Cradle to grave | Climate change (CO_2_eq): ±10,000kg |
|  |  | D: stainless steel scissors | 1 |  |  | Climate change (CO_2_eq): ±6,000kg |
|  |  | R: stainless steel scissors | 750 |  |  | Climate change (CO_2_eq): ±400kg |
| Rizan et al. ^9^ | LC | D: trocars, scissor, clip applier and cartridge | 1 | Hybrid  (ISO 14044) | Cradle to grave | Climate change (CO_2_eq): 7.19kg  Ecosystem quality^I^ (PDF*m2*yr): 4.36e^-5^  Human health^II^ (DALY/person/yr): 6.45e^-4^  Resources (US$): 2.7e^-5^ |
|  |  | R: trocars, scissor, clip applier and cartridge | 500 |  |  | Climate change (CO_2_eq): 1.76kg  Ecosystem quality^I^ (PDF*m2*yr): 1.04e^-5^  Human health^II^ (DALY/person/yr): 1.7e^-6^  Resources (US$): 5.82e^-6^ |
| Rizan et al. ^41^ | LC | D: trocars, scissors and clip applier | 1 | Hybrid (Greenhouse Gas protocol Product Life Cycle Accounting and Reporting Standard) | Cradle to factory gate | Climate change (CO_2_eq):   - 5mm trocar: 0.65kg - 12mm trocar: 1.08kg - Scissors: 0.55kg - Clip applier: 1.59kg |
|  |  | R: laparoscope, forceps, retractor | NA |  |  | Climate change (CO_2_eq):   - Laparoscope: 0.24kg - Forceps - Kelly crocodile grasping: 0.23kg - Forceps - Raptor toothed grasping: 0.15kg - Forceps - Rampley sponge holder: 0.13kg - Retractor: 0.17kg |
| *Gynecological surgery (n=1)* | | | | | | |
| Thiel et al. ^8^ | LH | D: laparoscopic instruments | 1 | Hybrid (NA) | NA | Climate change (CO_2_eq): 146kg |
|  |  | R: laparoscopic instruments | NA |  |  | Climate change (CO_2_eq): 36kg |
| *Pediatric surgery (n=1)* | | | | | | |
| Graham et al. ^52^ | SIPESA | D: stapler | NA | NA | NA | Waste production per case (kg)   - Metal: 0.582 - Paper: 0.012 - Plastics: 0.381 |
|  |  | R: clips with applier | NA |  |  | Waste production per case (kg)   - Paper: 0.009 - Plastics: 0.009 |

***Abbreviations:*** CED = Cumulative Energy Demand; CO2eq = CO2 equivalent (kg); DALY = Disability-Adjusted Life Years; D = Disposable; EIO = Economic Input-Output; kg = kilograms; kWh = kilowatt-hour; L = liters; LC = Laparoscopic Cholecystectomy; LCA = Life-Cycle Assessments; LH = Laparoscopic Hysterectomy; MJ = Megajoule; NA = Not Available; PDF*m2*yr = potentially disappeared fraction of species on the same surface per year; R = Reusable; RYGB = Roux-en-Y Gastric Bypass; SG = Sleeve Gastrectomy; SIPESA = Single-Incision Pediatric Endosurgery Appendectomy; TMR = Total Material Requirement (kg); USD = US Dollars ($); WRE = World ReCiPe Endpoint

^I^ Potentially disappeared fraction of species over a certain area during a certain time

^II^ Disability adjusted life years per person and year

^III^ Total mass of primary materials extracted from nature to support human activities

**Table B2.1: Cost outcomes of studies comparing disposables with reusables (n=20)**

| ***Author*** | ***Year*** | ***Procedure*** | ***Included instruments*** | ***No. of reuses*** | ***Cost per case*** | ***Cost reduction*** | ***Factors included in cost-estimation*** | | | |
| --- | --- | --- | --- | --- | --- | --- | --- | --- | --- | --- |
|  |  |  |  |  |  |  | *Purchase* | *Associated costs^1^* | *Processing costs^2^* | *Disposal^3^* |
| *Bariatric surgery (n=1)* | | | | | | | | | | |
| Yung et al. ^44^ | 2010 | LBS | D: ultrasonic shears | 1 | $56·97 | 29·30% | + | - | + | + |
|  |  |  | R: ultrasonic shears | 22 | $40·28 |  |  |  |  |  |
| *General surgery (n=12)* | | | | | | | | | | |
| Adler et al. ^18^ | 2005 | LC | D: trocars, scissors and Veress cannula | 1 | €449·16 | 94·67% | + | + | + | + |
|  |  |  | R: all other instruments | 255 | €23·96 |  |  |  |  |  |
| Apelgren et al. ^20^ | 1993 | LC | D: Veress needle, trocars, reducers, clip appliers with package of clips | 1 | $385·28 - $515·48 | 86·77%-90·89% | - | + | + | - |
|  |  |  | R: Veress needle, trocars, reducers, clip appliers with package of clips | 100 | $46·92 - $50·67 |  |  |  |  |  |
| Boberg et al. ^22^ | 2022 | LC | D: trocars | 1 | €75·13 | 42·01% | + | + | + | + |
|  |  |  | R: trocars | 250 | €42·57 |  |  |  |  |  |
| Demoulin et al. ^23^ | 1996 | LC | D: Veress needle, trocars, reducer, scissors, dissector, graspers, clip-applier with clips, suction, L-hook electrode | 1 | €1·31  (52·68 BEF) | 93·89% | + | + | + | + |
|  |  |  | R: Veress needle, trocars, reducer, scissors, dissector, graspers, clip-applier with clips, suction, L-hook electrode | 200 | €0·08 (3·06 BEF) |  |  |  |  |  |
| DesCôteaux et al. ^24^ | 1998 | GLP | limited-reusable scissors | 13 | $10 | 79·42% | + | + | + | - |
|  |  |  | reusable scissors | 25 | $48·60 |  |  |  |  |  |
|  |  |  | limited-reusable hook cautery | 17 | $5·18 | 0·58% |  |  |  |  |
|  |  |  | reusable hook cautery | 50 | $5·15 |  |  |  |  |  |
| Eddie et al. ^25^ | 1996 | GLP | D: trocars, scissors clip applicators and graspers | 1 | $381-710 | 76·38%-87·32% | + | + | + | + |
|  |  |  | R: trocars, scissors clip applicators and graspers | NA | $90 |  |  |  |  |  |
| Fengler et al. ^26^ | 1998 | GLP | D: scissors, forceps, trocars, Veress needle | 1 | DM 72·90 | 93·19% | + | + | - | - |
|  |  |  | R: scissors, forceps, trocars, Veress needle | 100 | DM 1070 |  |  |  |  |  |
| Ibbotson et al. ^29^ | 2013 | NA | D: plastic scissors | 1 | € 3·13 | 44·41% | + | + | + | + |
|  |  |  | D: stainless steel scissors | 1 | € 2·75 |  |  |  |  |  |
|  |  |  | R: stainless steel scissors | 750 | € 1·74 |  |  |  |  |  |
| MacFadyen et al. ^32^ | 1994 | LC | D: surgical instruments | 1 | $806 | 37·59% | + | + | + | + |
|  |  |  | R: surgical instruments | NA | $503 |  |  |  |  |  |
| Manatakis et al. ^34^ | 2014 | GLP | D: monopolar dissector, monopolar forceps, Metzenbaum scissors, suction, Veress needle, Hasson cannula and trocars | 1 | €305·27 | 89·22% | + | + | + | + |
|  |  |  | R: monopolar dissector, monopolar forceps, Metzenbaum scissors, suction, Veress needle, Hasson cannula and trocars | NA | €32·91 |  |  |  |  |  |
| Rizan et al. ^9^ | 2022 | LC | D: trocars, scissor, clip applier and cartridge | 1 | £282·17 | 53·75% | + | + | + | + |
|  |  |  | R: trocars, scissor, clip applier and cartridge | 500 | £130·50 |  |  |  |  |  |
| Slater et al. ^42^ | 2009 | LC | D: triocars, clip applicator with hemo-o-lok clips, Veress needle | 1 | £318·75 | 84·37% | + | + | + | + |
|  |  |  | R: trocars, clip applicator with hemo-o-lok clips, Veress needle | NA | £49·83 |  |  |  |  |  |
| *Gynecological surgery (n=5)* | | | | | | | | | | |
| Hasanov et al. ^45^ | 2017 | LH | D: Ligasure | 1 | €420 | 56·67% | + | - | - | + |
|  |  |  | R: Marseal | 50 | €182 |  |  |  |  |  |
| Holloran-Schwartz et al. ^46^ | 2016 | LH | D: Ligasure | 1 | $630·76 | 95·21% | + | + | + | + |
|  |  |  | R: bipolar forceps with monopolar scissors | 100 | $30·21 |  |  |  |  |  |
| Martínez-Zamora et al. ^47^ | 2009 | LH & LM | D: electric morcellator | 1 | €907·00 | 95·04% | + | - | - | - |
|  |  |  | R: electric morcellator | 300 | €45·00 |  |  |  |  |  |
| Ransom et al. ^48^ | 1996 | GL | D: 10mm infraumbilical cannula | 1 | $63·71 | 97·88% | + | + | - | + |
|  |  |  | R: 10mm infraumbilical cannula | 1000 | $1·35 |  |  |  |  |  |
| Schaer et al. ^50^ | 1995 | GL | D: optics, trocars, Veress needle converter, graspers, hook scissors, microscissors, suction | 1 | $252·29 | 87·17% | + | + | + | + |
|  |  |  | R: optics, Veress needle, trocars, probe | 3 year | $32·38 |  |  |  |  |  |
| *Pediatric surgery (n=2)* | | | | | | | | | | |
| Ebeid et al. ^51^ | 2016 | PLA | D: trocars | NA | NA | $300 (using reusables) | NA | NA | NA | NA |
|  |  |  | R: trocars | NA | NA |  |  |  |  |  |
| Graham et al. ^52^ | 2019 | SIPESA | D: stapler | NA | NA | $286-333 (using clips) | + | - | - | - |
|  |  |  | R: clips with applier | NA | NA |  |  |  |  |  |
|  |  |  | R: bipolar forceps with monopolar scissors | 100 | $30·21 |  |  |  |  |  |

***Abbreviations:*** € = Euro; £ = United Kingdom Pound; $ = United States Dollars; BEF = Belgian Franc; D = Disposable; DM = D Deutsche Mark; GL = Gynecologic Laparoscopy; GLP = General Laparoscopic Procedures; LBS = Laparoscopic Bariatric Surgery; LC = Laparoscopic Cholecystectomy; LH = Laparoscopic Hysterectomy; LM = Laparoscopic Myomectomy; NA = Not Available; PLA = Pediatric Laparoscopic Appendectomy; R = Reusable; SIPESA = Single-Incision Pediatric Endosurgery Appendectomy

+ = included in cost-estimation; - = not included in cost-estimation

^1^ Associated costs: instrument repair and replacement

^2^ Processing costs: labor expenditures incurred by materials management personnel to purchase, stock, store and distribute instruments
^3^ Disposal: waste disposal or cleaning and sterilization

**Table B2.2: Cost outcomes of studies comparing new with reprocessed disposables (n=3)**

| ***Author*** | ***Year*** | ***Procedure*** | ***Included instruments (no. of reuses)*** | ***Cost per case*** | ***Cost reduction*** | ***Factors included in cost-estimation*** | | | |
| --- | --- | --- | --- | --- | --- | --- | --- | --- | --- |
|  |  |  |  |  |  | *Purchase* | *Associated costs^1^* | *Processing costs^2^* | *Disposal^3^* |
| *General surgery (n=3)* | | | | | | | | | |
| Brady et al. ^54^ | 2017 | LCO | D: LigaSure (1) | $505 | 55·45% | *+* | *+* | *+* | *+* |
|  |  |  | RoD: LigaSure (NA) | $225 |  |  |  |  |  |
| Colak et al. ^58^ | 2004 | LC | D: trocars, dissectors, curved scissors, jaws, graspers, hooks, clips | $2680·23 | 56·12% | + | NA | + | NA |
|  |  |  | RDs: trocars (5), dissectors (7), curved scissors (5), jaws (15), graspers (5), hooks (10), clips (2) | $1176·17 |  |  |  |  |  |
| De Sousa Martins et al. ^60^ | 2018 | GLP | D: GIA stapler | €133·82 | 52%-56·62% | + | *+* | *+* | *-* |
|  |  |  | D: Harmonic | €512·43 |  |  |  |  |  |
|  |  |  | RDs: GIA stapler | €58·05 |  |  |  |  |  |
|  |  |  | RDs: Harmonic | €246 |  |  |  |  |  |

***Abbreviations:*** € = Euro; $ = United States Dollars; GLP = General Laparoscopic Practice; LC = Laparoscopic Cholecystectomy; LCO = Laparoscopic Colectomy; NA = Not Available; ND = ND = New Disposable; RDs = Reprocessed Disposable;

+ = included in cost-estimation; - = not included in cost-estimation

^1^ Associated costs: instrument repair and replacement

^2^ Processing costs: labor expenditures incurred by materials management personnel to purchase, stock, store and distribute instruments
^3^ Disposal: waste disposal or cleaning and sterilization

**Table B3.1: Performance outcome of studies comparing disposables with reusables (n=16)**

| ***Author*** | ***Procedure*** | ***Included instruments*** | ***No. of reuses*** | ***No. of participants or tests*** | ***Outcome*** | |
| --- | --- | --- | --- | --- | --- | --- |
| *Bariatric surgery (n=1)* | | | | | | |
| Yung et al. ^44^ | LBS | D: ultrasonic shears | 1 | 85 | OT (min) (mean, SD): 174 (15)  EBL (ml) (median, IQR): 83 (12) | P=0·34  P=0·06 |
|  |  | R: ultrasonic shears | 22 | 85 | OT (min) (mean, SD): 156 (15)  EBL (ml) (median, IQR): 63 (11) |  |
| *General surgery (n=8)* | | | | | | |
| DesCôteaux et al. ^24^ | GLP | limited-reusable scissors | 13 | 143 | Satisfaction rating^I^: 3·8 | NA |
|  |  | reusable scissors | 25 |  | Satisfaction rating^I^: 3·9 |  |
|  |  | limited-reusable hook cautery | 17 | 391 | Satisfaction rating^I^: 3·5 |  |
|  |  | reusable hook cautery | 50 |  | Satisfaction rating^I^: 4·4 |  |
| Harper et al. ^28^ | GLP | Direct Drive (Applied medical) | NA | 6 | Sum of ranking score^II^ (mean)   - Cutting: 4 - Ergonomics: 3·88   No· of imperfections per mm: 0·09 | NA |
|  |  | Endopath (Ethicon Endosurgery) | NA | 6 | Sum of ranking score^II^ (mean)   - Cutting: 2·63 - Ergonomics: 3   No. of imperfections per mm: 1·18 |  |
|  |  | Auto Suture Endo Shear (U.S. Surgocal) | NA | 6 | Sum of ranking score^II^ (mean)   - Cutting: 2·88 - Ergonomics: 2·63   No. of imperfections per mm: 1·09 |  |
| Kelty et al. ^30^ | GLP | D: trocar | 1 | 10 | Insertion force (PSI) (mean, SEM): 2·76 | NA |
|  |  | R: trocar | NA | 10 | Insertion force (PSI) (mean, SEM): 4·80 |  |
| Klar et al. ^31^ | GLP | D: LigaSure | 1 | 40 | Clamp pressure (N/mm^2^): 0·72  Sealing time (sec) (mean, SD): 9·10 (2·50)  Sealing zone (cm) (mean, SD): 0·43 (0·09)  Damage zone (cm) (mean, SD): 0·91 (0·13)  Seal failure: 6/40 (15%)  Burst pressures (mmHg) (mean, 95% CI): 484 (411-558) | NA  *P<0·001*  P=0·64  P=0·66  P=1·0  P=0·16 |
|  |  | R: MarSeal | NA | 40 | Clamp pressure (N/mm^2^): 0·77  Sealing time (sec) (mean, SD): 5·20 (1·1)  Sealing zone (cm) (mean, SD): 0·42 (0·08)  Damage zone (cm) (mean, SD): 0·93 (0·14)  Seal failure: 6/40 (15%)  Burst pressures (mmHg) (mean, 95% CI): 429 (395-462) |  |
| Mahmoud et al·^33^ | LC | D: Harmonic ultrasonic dissector | 1 | 30 | OT (min) (mean, SD): 28·02 (5·20)  Length of hospital stay (days) (mean, SD): 2·30 (1·77)  No. of cases converted to open: 1 (3,33%)  No. of cases with gallbladder perforation and bile leak: 6 (20%)  No. of major complications: 0 (0%)  No. of minor complications: 4 (13·33%) | *P=0·002*  *P=0·008* |
|  |  | R: monopolar electrosurgical energy dissector | NA | 30 | OT (min) (mean, SD): 39·94 (4·8)  Length of hospital stay (days) (mean, SD): 2·85 (1·43)  No. of cased converted to open: 3/30 (10%)  No. of cases with gallbladder perforation and bile leak: 10 (30%)  No. of major complications: 1 (3·33%)  No. of minor complications: 3 (10%) |  |
| Montero et al. ^35^ | GLP | D: L-hook | 1 | 61 | No. of instruments with insulation failure: 2 (3%) | *P<0*·*001* |
|  |  | R: laparoscopic instruments | NA | 165 | No. of instruments with insulation failure: 31 (19%) |  |
|  |  | R: instrument set | NA | 17 | No. of insulation failure in at least one instrument of the set: 12 (71%) |  |
| Nezhat et al. ^37^ | GLP | D: trocars | 1 | 50 | No. of complications^III^: 0  No. of failed insertions: 2 | P>0·05  P>0·05 |
|  |  | R: trocars | NA | 50 | No. of complications^III^: 3  No. of failed insertions: 4 |  |
| Richter et al. ^40^ | GLP | D: Ligasure | 1 | 35 | No. of instruments with seal failure: 3 (8·57%)  Desiccation^IV^ (mean, SEM): 1·91 (0·13)  Demarcation^V^ (mean, SEM): 1·82 (0·07)  Tissue charring^VI^ (mean, SEM): 1·30 (0·12)  Tissue clarity^VII^ (mean, SEM): 1·39 (0·20) | P=0·615  P=0·178  P=0·281  *P=0·050*  P=0·279 |
|  |  | R: BiClamp | NA | 36 | No. of instruments with seal failure: 1 (2·78%)  Desiccation^IV^ (mean, SEM): 2·17 (0·12)  Demarcation^V^ (mean, SEM): 1·67 (0·08)  Tissue charring^VI^ (mean, SEM): 1·64 (0·14)  Tissue clarity^VII^ (mean, SEM): 1·08 (0·18) |  |
| *Gynecological surgery (n=5)* | | | | | | |
| Hasanov et al. ^45^ | LH | D: Ligasure | 1 | 37 | OT (min) (mean, 95% CI): 22·7 (17·6-27·7)  EBL (ml) (median, IQR): 164 (110-217)  Postoperative fever (n, %): 0 (0%)  Overall postoperative complications (n, %): 1 (3%)  Length of hospital stay (days) (median, IQR): 4 (3-4) | P=0·89  P=0·36  NA  NA  NA |
|  |  | R: Marseal | 50 | 37 | OT (min) (mean, 95% CI): 26·4 (20-32·8)  EBL (ml) (median, IQR): 160 (116-203)  Postoperative fever (n, %): 1 (3%)  Overall postoperative complications (n, %): 0 (0%)  Length of hospital stay (days) (median, IQR): 4 (3-4) |  |
| Holloran-Schwartz et al. ^46^ | LH | D: Ligasure | 1 | 46 | Uterine vessel desiccation and electrosurgical cutting time (min) (median): 8·4 | *P<0·001* |
|  |  | R: bipolar forceps with monopolar scissors | 100 | 46 | Uterine vessel desiccation and electrosurgical cutting time (min) (median): 14·6 |  |
| Martínez-Zamora et al·^47^ | LH & LM | D: electric morcellator | 1 | 15 | MT (min) (median, IQR): 14·1 (2-17)  MR (g/min) (median, IQR): 28 (22-41)  MTW(g) (median, IQR): 410 (60-520)  Length of pieces (cm) (median, IQR): 4·6 (1-17) | *P<0·001*  *P<0·001*  P=0,86  *P<0·001* |
|  |  | R: electric morcellator | 300 | 14 | MT (min) (median, IQR): 11·3 (2-17)  MR (g/min) (median, IQR): 40·4 (26-47)  MTW(g) (median, IQR): 420 (57-550)  Length of pieces (cm) (median, IQR): 10·1 (1-43) |  |
| Ransom et al. ^48^ | GL | D: 10mm infraumbilical cannula | 1 | 529 | Bowel injury: 3/10.000 cases | *P<0*·*05* |
|  |  | R: 10mm infraumbilical cannula | 1000 | 9930 | Bowel injury: 19/10.000 cases |  |
| Rothmund et al·^49^ | LASH | D: EnSeal | 1 | 80 | OT (min) (mean, SD): 78·18 (33·96)  Length of hospital stay (days) (mean, SD): 2·01 (0·44) | *P=0·03*  *P=0·03* |
|  |  | R: bipolar coagulation device | NA | 80 | OT (min) (mean, SD): 86·31 (35·34)  Length of hospital stay (days) (mean, SD): 2·17 (0·47) |  |
| *Pediatric surgery (n=1)* | | | | | | |
| Graham et al. ^52^ | SIPESA | D: stapler | NA | 111 | OT (min) (mean, SD): 49·5 (16·9)  EBL (ml) (median, IQR): 4·66 (2·95-7·89)  Length of hospital stay (days) (median, IQR): 0·9 (0·3-1·7)  No. of complications (%): 11 (9·9) | P=0·91  P=0·43  P=0·43  P=0·25 |
|  |  | R: clips with applier | NA | 135 | OT (min) (mean, SD): 49·2 (16·3)  EBL (ml) (median, IQR): 4·85 (3·56-7·78)  Length of hospital stay (days) (median, IQR): 0·8 (0·2-1·8)  No. of complications (%): 20 (14·8) |  |
| *Thoracic surgery (n=1)* | | | | | | |
| Weksler et al. ^53^ | ETS | D: ultrasonic scalpel | 1 | 70 | Length of hospital stay (hours) (mean, SD): 11·4 (5·9)  No. of complications: 13 | P=0·24 |
|  |  | R: monopolar hook cautery | NA | 70 | Length of hospital stay (hours) (mean, SD): 10·1 (5·4)  No. of complications: 8 |  |

***Abbreviations:*** D = Disposable; EBL = Estimated Blood Loss; ETS = Endoscopic Thoracic Sympathectomy; GL = Gynecology Laparoscopy; GLP = General Laparoscopic Procedures; LASH = Laparoscopic Supracervical Hysterectomy; LBS = Laparoscopic Bariatric Surgery; LH = Laparoscopic Hysterectomy; LM = Laparoscopic Myomectomy; NA = Not Available; MR = Morcellating Rate; MT = Morcellating Time; MTW = Morcellating Tissue Weight; OT = Operating Time; PSI = Pounds per Square Inch; R = Reusable; SEM = Standard Error of Mean; SIPESA = Single-Incision Pediatric Endosurgery Appendectomy.

^I^ Not further defined

^II^ Higher rating score equals more desirable

^III^ Omental perforation, subcutaneous emphysema

^IV^ 0 = wet, 1 = slightly dried, 2 = moderately dried, 3 = fully dried

^V^ 0 = sealed tissue visually not to different from normal tissue, 1 = only slightly different, 2 = moderately different, 3 = sharply demarcate

^VI^ 0 = no charring, 1 = few black spots, 2 = confluent black spots, 3 = complete black area

^VII^ 0 = not translucent, 1 = slightly translucent, 2 = moderate translucent, 3 = fully translucent

**Table B3.2: Performance outcomes of studies comparing new with reprocessed disposables (n=9)**

| ***Author*** | ***Procedure*** | ***Included instruments (no. of uses)*** | ***Cleaning method*** | ***No. of participants or tests*** | ***Outcome*** | |
| --- | --- | --- | --- | --- | --- | --- |
| *General surgery (n=9)* | | | | | | |
| Brady et al. ^54^ | LCO | ND: LigaSure (1) | NA | 76 | OT (min) (mean, SD): 131 (48)  EBL (ml) (mean, SD): 20 (25)  No. of pedicle bleedings requiring intervention: 4 (5·2%)  Length of hospital stay (days) (mean, SD): 3·8 (0·5)  No. of reoperations: 1 (1·3%) | P=0·47  P=0·18  P=0·12  P=0·18  P=1·0 |
|  |  | RD: LigaSure (NA) |  | 76 | OT (min) (mean, SD): 128 (41)  EBL (ml) (mean, SD): 25 (15)  No. of pedicle bleedings requiring intervention: 3 (3,9%)  Length of hospital stay (days) (mean, SD): 3,5 (0·6%)  No. of reoperations: 1 (1·3%) |  |
| Carungi et al. ^55^ | GLP | ND: Harmonic scalpels (1) | Ethylene oxide | 30  3 | No. of instruments with 300 activations completed: 22/30 (73%)  Time to transection – vessel (sec) (mean): 2·7  Time to transection – liver (sec) (mean): 3·4  Time to achieve hemostasis – vessel (sec) (mean): 0  Time to achieve hemostasis – liver (sec) (mean): 43·8 | *P=0,005*  P=0·182  P=0·398  P=1·0  P=0·541 |
|  |  | RD: Harmonic scalpels (NA) |  | 30  3 | No. of instruments with 300 activations completed: 30/30 (100%)  Time to transection – vessel (sec) (mean): 2·2  Time to transection – liver (sec) (mean): 4·1  Time to achieve hemostasis – vessel (sec) (mean): 0  Time to achieve hemostasis – liver (sec) (mean): 88·1 |  |
| Colak et al. ^58^ | LC | ND: trocars (1), dissectors (1), curved scissors (1), jaws (1), graspers (1), hooks (1), clips (1) | Alkalinized 2%  glutaraldehyde | 62 | OT (min) (mean, SD): 42·66 (13·92)  Length of hospital stay (days) (mean, SD): 1·53 (2·61)  No. of trocar-side infections (mean, %): 1 (1,6%)  Conversion rate (mean, %): 1 (1,6%) | P=0·65  P=0·50  P=0·57  P=0·57 |
|  |  | RD: trocars (5), dissectors (7), curved scissors (5), jaws (15), graspers (5), hooks (10), clips (2) |  | 63 | OT (min) (mean, SD): 43,88 (16,29)  Length of hospital stay (days) (mean, SD): 1,30 (0,75)  No. of trocar-side infections (mean, %): 2 (3·2%)  Conversion rate (mean, %): 2 (3·2%) |  |
| De Sousa Martins et al. ^60^ | GLP | ND: GIA stapler and Harmonic (NA) | NA | 417 | OT (hours) (mean, SD): 2·45 (1·52)  Length of hospital stay (days) (mean, SD): 10·36 (12·00)  Infection rate: 13·4%  No. of re-hospitalizations: 33 (7·91%) | P=0·161  P=0·881  NA P=0·678 |
|  |  | RD: GIA stapler and Harmonic (NA) |  | 316 | OT (hours) (mean, SD): 2,33 (1,53)  Length of hospital stay (days) (mean, SD): 9,55 (8,92)  Infection rate: 12,3%  No. of re-hospitalizations: 28 (8,86%) |  |
| Gärtner et al. ^62^ | GLP | ND: ultrasonic scissors (1) | NA | 51 | No. of instruments with:   - Optimal force of activation: 48/51 (94·12%) - Optimal cutting effect: 44/51 (86·27%) - Optimal coagulation effect: 45/51 (88·23%) | P=1·0  P=1·0  P=1·0 |
|  |  | RD: ultrasonic scissors (NA) |  | 49 | No. of instruments with:   - Optimal force of activation: 47/49 (95·92%) - Optimal cutting effect: 43/44 (87·75%) - Optimal coagulation effect: 43/44 (87·75%) |  |
| Lester et al·^65^ | GLP | ND: Harmonic scalpel (1) | NA | 6 | Peak temperature (°C) (mean, SD): 48,4 (8·2)  Time to peak temperature (sec) (mean, SD): 11,6 (1·5)  Transection duration (sec) (mean, SD): 3·40 (0·98) | P=0·623  P=0·598  NA |
|  |  | RD: Harmonic scalpel (2-4) |  | 6 | Peak temperature (°C) (mean, SD): 48,3 (7·2)  Time to peak temperature (sec) (mean, SD): 11,4 (1·6)  Transection duration (sec) (mean, SD): NA |  |
|  |  | RD: Harmonic scalpel (3) |  | 6 | Peak temperature (°C) (mean, SD): 48,3 (6·7)  Time to peak temperature (sec) (mean, SD): 11,3 (1·2)  Transection duration (sec) (mean, SD): 3·27 (0.84) |  |
| Mihanovic et al. ^66^ | LA | ND: Harmonic scalpel (1) | Sekusept^TM^ enzymatic detergent | 49 | OT (hours) (median, IQR): 22 (20-30)  Length of hospital stay (days) (median, IQR): 2 (2-3)  No. of intraoperative complications: 0 (0%)  No. of postoperative complications: 2 (4·1%)  No. of re-operations: 0 (0%)  No. of surgeons assessing the parameter as optimal:   - Hemostasis: 48 (98%) - Coagulation efficiency: 48 (98%) - Cutting efficiency: 47 (95·90%) - Force applied: 47 (95·90%) - Error messages: 49 (100%) | P=0·233  P=0·998  NA  P=0·536  NA  P=0·281  P=0·112  P=0·678  P=0·999  P=0·057 |
|  |  | RD: Harmonic scalpel (2) |  | 51 | OT (hours) (median, IQR): 25 (21-35)  Length of hospital stay (days) (median, IQR): 2 (2-3)  No. of intraoperative complications: 0 (0%)  No. of postoperative complications: 1 (2%)  No. of re-operations: 0 (0%)  No. of surgeons assessing the parameter as optimal:   - Hemostasis: 46 (90·2%) - Coagulation efficiency: 45 (88·20%) - Cutting efficiency: 46 (90·20%) - Force applied: 48 (94·10%) - Error messages: 46 (90·20%) |  |
| Mues et al. ^67^ | GLP | ND: 5mm bladed trocars and 5mm bladeless trocar | NA | 199 | No. of instruments with damage: 5/132 (3·79%)  Mean insertion force (pound per force):   - B5LT trocar: 9·18 - D5LT trocar: 7·18 - D12LT trocar: 8·91   Mean removal force (pound per force):   - B5LT trocar: 9,63 - D5LT trocar: 7,27 - D12LT trocar: 9,39   Mean leak rate (mL/min):   - Before probe insertion: 78,5 - After probe insertion: 89,3 | P=0·063  P=0·237  P=0·993  *P=0·021*  P=0·984  *P=0·004*  P=0·167  *P=0·0001*  *P=0·0001* |
|  |  | RD: 5mm bladed trocars and 5mm bladeless trocar |  | 328 | No. of instruments with damage: 73/259 (28·2%)  Mean insertion force (pound per force):   - B5LT trocar: 8·62 - D5LT trocar: 8·14 - D12LT trocar: 11·13   Mean removal force (pound per force):   - B5LT trocar: 8·66 - D5LT trocar: 6·97 - D12LT trocar: 10·37   Mean leak rate (mL/min):   - Before probe insertion: 169·0 - After probe insertion: 161·2 |  |
| Weld et al. ^68^ | GLP | ND: Ethicon Shears LCS-C5 (1) |  | 90 | No. of instruments with visual abnormality:   - Blade buffing: 0 (0%) - Blade scratches: 2 (2%) - Blade nicks: 0 (0%) - Blent blades: 0 (0%) - Bent drive tabs: 0 (0%) - Bent horse collar: 11 (12%) - Cracked horse collar: 0 (0%) - Pad debris: 14 (16%) - Visible glue: 0 (0%) - Metallic burrs: 0 (0%) - Pad separation: 12 (13%) - Shroud overlap: 3 (3%)   Mean dislodge force (lb): 5,8 (4,7-7,4)  Mean leak rate (mL/min): 6 (5-6)  Mean clamp force (pound): 1,3 (1,1-1,5)  Mean power (Watt): 5,17 (4,27-6,83) | *P<0·01*  *P<0·01*  *P<0·01*  *P<0·01*  *P<0·01*  *P<0·01*  *P=0·02*  *P<0·01*  *P<0·01*  *P=0·02*  P=0·52  *P<0·01*  *P<0*·*01*  P=0·76  P=0·50  P=0·10 |
|  |  | RD: Ethicon Shears LCS-C5 (150) |  | 89 | No. of instruments with visual abnormality:   - Blade buffing: 71 (80%) - Blade scratches: 16 (18%) - Blade nicks: 25 (28%) - Blent blades: 13 (14%) - Bent drive tabs: 36 (40%) - Bent horse collar: 36 (40%) - Cracked horse collar: 5 (6%) - Pad debris: 70 (79%) - Visible glue: 65 (73%) - Metallic burrs: 5 (6%) - Pad separation: 14 (16%) - Shroud overlap: 3 (3%)   Mean dislodge force (lb): 4,4 (2,7-6,0)  Mean leak rate (mL/min): 6 (5-9)  Mean clamp force (pound): 1,3 (1,2-1,7)  Mean power (Watt): 5,27 (4,45-6,36) |  |

***Abbreviations:*** EBL = Estimated Blood Loss; GLP = General Laparoscopic Practice; LA = Laparoscopic Appendectomy; LC = Laparoscopic Cholecystectomy; LCO = Laparoscopic Colectomy; NA = Not Available; ND = New Disposable; OT = Operating Time; RDs = Reprocessed Disposable

**Table B4.1: Contamination risk outcomes of studies on reusable instruments (n=6)**

| ***Author*** | ***Year*** | ***Type of contamination*** | ***Cleaning method*** | ***Detergent (exposure time)*** | ***Evaluation method of residuals*** | ***Instruments*** | ***No. of tests*** | ***Outcome*** |
| --- | --- | --- | --- | --- | --- | --- | --- | --- |
| *General surgery (n=6)* | | | | | | | | |
| Alfa et al. ^19^ | 2003 | Artificial test soil (protein, carbohydrate, hemoglobin and); Enterococcus faecalis; Geobacillus stearothermophilus | SI-Auto Narrow lumen cleaner;  Manual cleaning | 3E-zyme (5 min) | Quantitative analysis (protein, carbohydrate, haemoglobin, viable counts); Bradford reagent (protein); TMB-One reagent (haemglobin) | Ported instruments | NA | Reduction in soil parameters  SI-Auto:   - Protein volume: 99·9% - Carbohydrate volume: 99·5% - Haemoglobin: 100%   Manual cleaning:   - Protein volume: 49·8% - Carbohydrate volume: 57·1% - Haemoglobin: 43·0% |
|  |  |  |  |  |  | Non-ported instruments | NA | Reduction in soil parameters  SI-Auto:   - Protein volume: 99·9% - Carbohydrate volume: 99·7% - Haemoglobin: 100%   Manual cleaning:   - Protein volume: 99·2% - Carbohydrate volume: 99·7% - Haemoglobin: 98·8% |
| Baxter et al. ^21^ | 2006 | Patient residuals | NA | NA | Scanning electron microscopy; energy dispersive X-ray spectroscopic anaylsis; hydrolysis of amino acids | Tray 1 | 8 | Residual proteins per instrument (median) (µg): 267 |
|  |  |  |  |  |  | Tray 2 | 27 | Residual proteins per instrument (median) (µg): 260 |
|  |  |  |  |  |  | Tray 3 | 48 | Residual proteins per instrument (median) (µg): 163 |
| Fengler et al. ^27^ | 2000 | Patient residuals | Ultrasonic bath; washer-disinfection | NA | Sodium dodecyl analysis; erythrocyte quick-test sticks | Laparoscopic tray | NA | No. of instruments with residual proteins: 8/32 (25%) No. of instruments with positive hemoglobin pseudoperoxidase test stick: 4/32 (12·5%) |
| Murdoch et al. ^36^ | 2006 | Patient residuals | Autoclave | NA | BioRad-detergent-compatible protein assay; o-phthaldialdehyde/N-acetyl cysteine  o-phthaldialdehyde/N-acetyl cysteine | Retractors | 23 | No. of instruments with residual proteins: 10/23 (43·47%) |
|  |  |  |  |  |  | Scissors | 28 | No. of instruments with residual proteins: 8/28 (28·57%) |
|  |  |  |  |  |  | Forceps | 29 | No. of instruments with residual proteins: 11/29 (37·93%) |
|  |  |  |  |  |  | Gags | 12 | No. of instruments with residual proteins: 6/12 (50%) |
|  |  |  |  |  |  | Birkett | 6 | No. of instruments with residual proteins: 4/6 (66·66%) |
|  |  |  |  |  |  | Dissectors | 5 | No. of instruments with residual proteins: 2/5 (40%) |
| Owusu et al. ^38^ | 2022 | Patient residuals | Autoclave or hot air oven | NA | Agar plates for bacteria culture; Bruker  MALDI Biotyper® IVD for Bacillus cereus and Staphylococcus hominis | Dissecting forceps | 5 | No. of bacteria isolates: 0/5 (0%) |
|  |  |  |  |  |  | Kocher forceps | 6 | No. of bacteria isolates: 3/6 (15,7%)  Type of bacteria isolate: Bacillus cereus |
|  |  |  |  |  |  | Rampley sponge holding forceps | 7 | No. of bacteria isolates: 0/7 (0%) |
|  |  |  |  |  |  | Curved mosquito artery forceps | 7 | No. of bacteria isolates: 0/7 (0%) |
|  |  |  |  |  |  | Metzenbaum scissors | 5 | No. of bacteria isolates: 0/5 (0%) |
|  |  |  |  |  |  | Mayo’s scissors | 8 | No. of bacteria isolates: 2/8 (10·5%)  Type of bacteria isolate: Bacillus cereus |
|  |  |  |  |  |  | Langenberg retractor | 7 | No. of bacteria isolates: 0/7 (0%) |
|  |  |  |  |  |  | Deaver retractor | 6 | No. of bacteria isolates: 4/6 (22·1%)  Type of bacteria isolate: Citrobacter freundii |
| Raina et al. ^39^ | 2018 | Patient residuals | Sterilisation | 2% lutaraldehyde solution (30 min); saline | PSI within 30 days of surgery | Laparoscopic instruments | 112 | No. of patients with superficial PSI: 4/112 (3·57%)  No. of patients with deep PSI: 2/112 (1·78%) |

***Abbreviations:*** NA = not available; PSI = Port-Site Infection

**Table B4.2: Contamination risk outcomes of studies comparing new with reprocessed disposables (n=6)**

| ***Author*** | ***Year*** | ***Type of contamination*** | ***Cleaning method*** | ***Detergent (exposure time)*** | ***Evaluation method of residuals*** | ***Instruments (no. of reuses)*** | ***No. of tests*** | ***Outcome*** | |
| --- | --- | --- | --- | --- | --- | --- | --- | --- | --- |
| *General surgery (n=6)* | | | | | | | | | |
| Chan et al. ^56^ | 2000 | Preinfected horse blood containing polio type 1 virus or herpes simplex virus type 1 (HSV) | Ultrasonic bath | Haemo-Sol solution (20 min) | Cultures | RD: 10/11mm trocars (NA) | 4 | No. of positive cultures for polio: 2/4 (50%)  No. of positive cultures for HSV: 0/4 (0%) |  |
| Chivukula et al. ^57^ | 2020 | Patient residuals | Water and 70% ethanol | NA | Visual inspection; optical microscopy; SEM; hemoglobin detection | ND: LigaSure (1) | 84 | No. of instruments with foreign materials:   - Visual inspection: 0/84 (0%) - Optical microscopy: 0/12 (0%) - SEM: 0/12 (0%) - Haemoglobin detection: 0/24 (0%) | NA |
|  |  |  |  |  |  | RD: LigaSure (NA) | 84 | No. of instruments with foreign materials:   - Visual inspection: 44/84 (52%) - Optical microscopy: 12/12 (100%) - SEM: 11/12 (92%) - Haemoglobin detection: 6/12 (50%) |  |
| De Lion Botero Couto Lopes et al. ^59^ | 2011 | Geobacillus stearothermophilus | Ultrasonic bath and manual cleaning | Amylase, protease, lipase, carbohydrase, nonionic surfactants and isopropyl alcohol (30 min) | Counting CFU per unit of material | R: grasper (NA), curved scissors (NA), curved dissector (NA), electrosurgery probe (NA), Veress needle (NA) | 35 | CFU/material: 0 | NA |
|  |  | 10^6^ CFU *Bacillus atrophaeus var. niger* | Ethylene oxide, and autoclave | NA |  | RD: grasper (NA), curved scissors (NA), curved dissector (NA), electrosurgery probe (NA), Veress needle (NA) | 95 | CFU/material: 0 |  |
| Dos Santos et al. ^61^ | 2008 | Patient residuals | Sterilisation (ethylene oxide, steam sterilisation or hydrogen peroxide plasma) | Saline (NA); Endozime (NA); Tyvek (NA) | Cultures | RD: 5mm, 11mm and 12mm trocars (NA) | 28 | No. of positive cultures before sterilisation: 13/28 (4643%)  No. of positive cultures after sterilisation: 0/28 (0%) |  |
| Gundogdu et al. ^63^ | 1998 | Patient residuals | NA | Alkalinized 2% glutaraldehyde (15 min.) | Cultures (8/instrument) | ND: trocars (1) | 15 | No. of positive cultures: 0/15 | P>0·05 |
|  |  |  |  |  |  | RD: trocars (NA) | 30 | No. of positive cultures: 1/30 (3,33%)  Type of bacteria isolate: gram-positive bacilli |  |
| Jokar et al. ^64^ | 2022 | Patient residuals | NA | NA | NA | ND: 5mm and 11 mm trocars (1) | 215 | No. of patients with PSI:   - 3-5 days after surgery: 4/215 (1·9%) - 5-14 days after surgery: 0/215 (0%) - 3-14 days after surgery: 4/215 (1·9%) | *P=0·013*  *P=0·018*  *P=0·002* |
|  |  |  |  |  |  | RD: 5mm and 11 mm trocars (NA) | 258 | No. of patients with PSI:   - 3-5 days after surgery: 17/258 (6·6%) - 5-14 days after surgery: 7/258 (2·7%) - 3-14 days after surgery: 22/258 (8·5%) |  |

***Abbreviations:*** DLIs = Disposable Laparoscopic Instruments; GLP = General Laparoscopic Practice; IVS = In-Vitro Study; LC = Laparoscopic Cholecystectomy; LM = Light Microscopy; ND = New Disposable; PS = Prospective Study; PSI = Port Site Infection; R = Reusable; RDs = Reprocessed Disposable; RNM = Radionuclide Method; SEM = Scanning Electron Microscopy; XPS = X-ray Photoelectron Spectroscopy
